# Supplementary material for: Hepatitis B vaccination coverage, knowledge and sociodemographic determinants of uptake in high risk public safety workers in Kaduna State, Nigeria: a cross sectional survey
Source: BMJ Open. 2017 Jun 2;7(5):e015845. doi: 10.1136/bmjopen-2017-015845 (PMC5541342; doi:10.1136/bmjopen-2017-015845)
Supplement: Supplementary data [file bmjopen-2017-015845supp001.pdf]

APPENDIX A: ADAPTED STUDY QUESTIONNAIRE

HEPATITIS B VACCINATION QUESTIONNAIRE

For Official Use Only

Researcher…………………………………. Questionnaire no: ☐

Date of Data Collection (DD/MM/YY)……………………….

IMPLIED CONSENT (Please read before completing questionnaire): Having gone through the research information contained in the participant information sheet, by completing this questionnaire you are consenting to participate in the study. If you do not wish to complete the questionnaire, please put the blank version into the envelope and I will collect it with all other questionnaires. Thank you.

Please only complete the questionnaire if you are aged 18 years and above and have at least 6 months of service with FRSC

Note: This questionnaire is anonymous; please do not write your name on it. Kindly give an answer to all the questions as it pertains to you and please answer as truthfully as you can.

Please check (✔) only the box that most correctly answers the question, making sure you make only one selection for each question except where otherwise indicated.

Section A: Demographic Questions

1. What is your sex?

☐ Male

☐ Female

2. What was your age on your last birthday?

- ☐ 18 to 29 years
- ☐ 30 to 39 years
- ☐ 40 to 49 years
- ☐ 50 years and above

Section B: Employment History

3. How long have you worked with Federal Road Safety Corps (FRSC)?

- ☐ 6 months to 2 years
- ☐ 3 years to 10 years
- ☐ 11 years to 19 years
- ☐ 20 years and above

4. What is your cadre?

- ☐ Officer
- ☐ Marshal Inspectorate
- ☐ Road Marshal Assistant

5. Please check (✔) the box below your rank

ACM	CC	DCC	ACC	CRC	SRC	RC	DRC	ARC

CI	DCI	ACI	PMI	SMI	MI-I	MI-II	MI-III	CRMA	DCRMA	SRMA	RMAI	RMAII	RMAIII

Section C: Perception of Risk of Exposure to Hepatitis B Virus

6. Have you ever heard about hepatitis B virus infection?

☐ Yes

☐ No

7. How serious do you think being infected with hepatitis B virus is compared to HIV?

☐ Less serious than HIV

☐ As serious as HIV

☐ More serious than HIV

☐ I don't know

8. How can someone be infected with hepatitis B virus? (please check (✔) all the correct boxes if your answer is more than one)

☐ Through contact with blood of an infected person

☐ Through contact with saliva of an infected person

☐ Through contact with sweat of an infected person

☐ Through contact with body fluid contaminated by blood of an infected person

☐ I don't know

9. How much do you think your work with FRSC exposes you to the risk of contracting hepatitis B virus infection?

- ☐ No risk of exposure
- ☐ Low risk of exposure
- ☐ Moderate risk of exposure
- ☐ High risk of exposure
- ☐ I don't know

Section D: Hepatitis B vaccination Knowledge and Status

10. Have you ever heard about hepatitis B vaccination?

- ☐ Yes
- ☐ No

11. How effective do you think hepatitis B vaccination is in protecting someone against hepatitis B virus infection?

- ☐ Not effective
- ☐ Slightly effective
- ☐ Very effective
- ☐ I don't know

12. Have you ever received hepatitis B vaccination?

- ☐ Yes
- ☐ No

If your answer to question 12 is 'No', answer question 13; if it is 'Yes', go to question 14

13. Why have you not received hepatitis B vaccination? (Please check (✔) all the correct boxes if your answer is more than one)

- ☐ I am not aware of hepatitis B vaccination
- ☐ I do not know where to go and receive it
- ☐ I don't have time
- ☐ It is expensive
- ☐ I don't see the need
- ☐ I am afraid of contracting the virus from the vaccine
- ☐ Others (please state)……………………………………………………………………………

14. If your answer to question 12 is 'Yes', how many doses of hepatitis B vaccine have you received?

- ☐ 1 dose
- ☐ 2 doses
- ☐ 3 doses
- ☐ More than 3 doses

15. When did you receive the last dose of hepatitis B vaccine?

- ☐ Less than 1 month ago
- ☐ 1 month to 3 months ago
- ☐ 4 months to 6 months ago
- ☐ More than 6 months ago

16. What do you think is the recommended full dose of hepatitis B vaccine?

- ☐ 1 dose
- ☐ 2 doses
- ☐ 3 or more doses
- ☐ I don't know

17. How long does a full dose of hepatitis B vaccine protect someone?

- ☐ Less than 1 year
- ☐ 1 year to 5 years
- ☐ 6 years to 10 years
- ☐ 11 years to 19 years
- ☐ 20 years or more
- ☐ I don't know

Thank you for your time!

APPENDIX B: Knowledge Scoring Table

Scoring Table: HBV (A) and HBVc (B) Knowledge Questions and Scoring Pattern

**A. Question**	**Options checked**	**Score**
Have you heard about hepatitis B virus infection? (**one option**)	Yes (✔)<br><br>No (✔)	1<br><br>0
How serious do you think being infected with hepatitis B virus is compared to HIV? (**one option**)	Less serious than HIV (✔)<br><br>As serious as HIV (✔)<br><br>More serious than HIV (✔)<br><br>I don't know (✔)	0<br><br>0<br><br>1<br><br>0
How can someone be infected with hepatitis B virus? (please check (✔) all the correct boxes if your answer is more than one)	Through contact with blood of an infected person (✔)<br><br>Through contact with saliva of an infected person (**blank**)*<br><br>Through contact with sweat of an infected person (**blank**)*<br><br>Through contact with body fluid contaminated by blood of an infected person (✔)<br><br>I don't know (✔)	1<br><br>1<br><br>1<br><br>1<br><br>0 for all options
Maximum HBV knowledge Score<br>Minimum HBV knowledge Score		6 of 6<br>0 of 6
**B. Question**	**Options**	**Score**
Have you ever heard about hepatitis B vaccination? (**one option**)	Yes (✔)<br><br>No (✔)	1<br><br>0
How effective do you think hepatitis B vaccination is in protecting someone against hepatitis B virus infection? (**one option**)	Not effective (✔)<br><br>Slightly effective (✔)<br><br>Very effective (✔)<br><br>I don't know (✔)	0<br><br>0<br><br>1<br><br>0
What do you think is the recommended full dose of hepatitis B vaccine? (**one option**)	1 dose (✔)<br><br>2 doses (✔)<br><br>3 or more doses (✔)<br><br>I don't know (✔)	0<br><br>0<br><br>1<br><br>0
How long does a full dose of hepatitis B vaccine protect	Less than 1 year (✔)	0

A. Question	Options checked	Score
someone? (**one option**)	1 year to 5 years (✔)<br>6 years to 10 years (✔)<br>11 years to 19 years (✔)<br>20 years or more (✔)<br>I don't know (✔)	0<br>0<br>0<br>1<br>0
Maximum HBVc knowledge score<br>Minimum HBVc knowledge score		4 of 4<br>0 of 4

***** HBV can be found in saliva but the concentration is very low compared to blood; direct injection through bites is required to transmit the virus via this medium.[1,2] Transmission has not been observed through sweat.[1]

References:

1. Canadian Center for Occupational Health and Safety (CCOHS). Hepatitis B. *OSH Facts Sheet* 2014. http://www.ccohs.ca/oshanswers/diseases/hepatitis_b.html (Accessed: 27th July 2015).
2. Centers for Disease Control and Prevention (CDC). When Someone Close to You has Viral Hepatitis. *Division of Viral Hepatitis* 2010. http://www.cdc.gov/hepatitis/HBV/PDFs/HepBWhenSomeoneClose.pdf (Accessed: 26th June 2015).

Appendix C: Frequency of Available Data and Missing Data from Completed Questionnaires, Federal Road Safety Corps, Kaduna Sector Command, Nigeria, June-July, 2015

Variable	Valid Sample Size	Available Data	Percentage	Missing Data	Percentage
Sex	**341**	**327**	**95.9**	**14**	**4.1**
Age	**341**	**338**	**99.1**	**3**	**0.9**
Duration of Service	**341**	**339**	**99.4**	**2**	**0.6**
Cadre	**341**	**341**	**100**	**0**	**0**
Ever heard of HBV infection?	**341**	**340**	**99.7**	**1**	**0.3**
Seriousness of HBV compared to HIV	**341**	**335**	**98.2**	**6**	**1.8**
Route of Transmission of HBV	**341**	**337**	**98.8**	**4**	**1.2**
Perception of Risk of exposure to HBV	**341**	**340**	**99.7**	**1**	**0.3**
Ever heard of hepatitis B vaccination?	**341**	**336**	**98.5**	**5**	**1.5**
Effectiveness of hepatitis B vaccination	**341**	**334**	**97.9**	**7**	**2.1**
Ever received hepatitis B vaccination?	**341**	**325**	**95.3**	**16**	**4.7**
Number of doses received	**198**	**198**	**100**	**0**	**0**
Recommended full dose of hepatitis B vaccine	**341**	**308**	**90.3**	**33**	**9.7**
Duration of protection from full-dose HBVc	**341**	**312**	**91.5**	**29**	**8.5**
