# Supplementary material for: Hepatitis B vaccination coverage, knowledge and sociodemographic determinants of uptake in high risk public safety workers in Kaduna State, Nigeria: a cross sectional survey
Source: BMJ Open. 2017 Jun 2;7(5):e015845. doi: 10.1136/bmjopen-2017-015845 (PMC5541342; doi:10.1136/bmjopen-2017-015845)
Supplement: Author's manuscript [file bmjopen-2017-015845.draft_revisions.pdf]

BMJ Open

Survey of Hepatitis B Vaccination Coverage, and Knowledge and Socio-Demographic Determinants of Uptake in Members of the Federal Road Safety Corps, Kaduna State, Nigeria

Journal:	*BMJ Open*
Manuscript ID	bmjopen-2017-015845
Article Type:	Research
Date Submitted by the Author:	03-Jan-2017
Complete List of Authors:	Ochu, Chinwe; Ahmadu Bello University Teaching Hospital, Family Medicine<br>Beynon, Caryl; University of Liverpool
**Primary Subject Heading**:	Public health
Secondary Subject Heading:	Epidemiology, Occupational and environmental medicine, Infectious diseases
Keywords:	Infection control < INFECTIOUS DISEASES, Hepatitis B virus, Vaccination coverage, Public safety workers

SCHOLARONE™
Manuscripts

For peer review only

Survey of Hepatitis B Vaccination Coverage, and Knowledge and Socio-Demographic Determinants of Uptake in Members of the Federal Road Safety Corps, Kaduna State, Nigeria

Corresponding Author: Ochu, Chinwe Lucia

Department: Family Medicine

Institution: Ahmadu Bello University Teaching Hospital

City: Zaria

State: Kaduna State

Postal code: 810105

Country: Nigeria

Emails: lucia.ochu@online.liverpool.ac.uk; luciaochu@yahoo.com

Phone: +2348027413202

Co-Author: Beynon, Caryl M.

Department: Public Health

Institution: University of Liverpool (online)

Country: United Kingdom

Email: caryl.beynon@online.liverpool.ac.uk

Phone: NA

Word Counts: Abstract: 295; Main text: 3956 (excluding title page, statements, abstract, article summary, tables, acknowledgment, text box and references).

Number of tables: 5

Number of references: 39

Number of supplementary files (appendices) for online only publication: 3

For peer review only

STATEMENTS

Contributorship: This study was carried out as a dissertation research by CLO under the close supervision of CMB, in partial fulfillment of the requirement for the award of the degree of Master of Public Health by the University of Liverpool, United Kingdom. CLO collected the data and conducted the analyses. These were reviewed by CMB. The manuscript was drafted by CLO and reviewed and revised by CMB. Both authors approved the final version for publication.

Competing Interests: None declared.

Funding: This research received no specific grant from any funding agency in the public, commercial or not-for-profit sectors.

Ethics Approval: This study was approved by the University of Liverpool's Ethics Committee and the Ethics Committee of Kaduna State Ministry of Health.

Participant Consent: Obtained

Additional Data: The table showing the frequency of available and missing data for the study variables is uploaded as a supplementary document. Dataset is available at Dryad data repository via this link:

For peer review only

ABSTRACT

Objectives: To estimate hepatitis B vaccination coverage, and knowledge and socio-demographic determinants of full-dose uptake in Federal Road Safety Corps (FRSC) members, Kaduna State, Nigeria in order to inform relevant targeted vaccination policies.

Design: A cross-sectional survey of FRSC members, Kaduna Sector Command.

Settings: Six randomly-selected Unit Commands under Kaduna Sector Command, Kaduna State, Nigeria.

Participants: Pilot-tested structured self-administered questionnaire was administered to 341 participants aged 18 years and above with ≥6 months of service between 17th June and 22nd July, 2015. Excluded were FRSC members in RS1 Zonal Command headquarters as the Zonal Command includes other States beyond the study scope.

Primary Outcome: Hepatitis B vaccination status of participants categorized as 'not vaccinated' for uptake of <3 doses and 'vaccinated' for uptake of ≥3 doses.

Analysis: Descriptive analysis estimated hepatitis B vaccination coverage while logistic regression ascertained associations.

Results: Any dose hepatitis B vaccination coverage was 60.9%; full-dose coverage was 30.5%. Less than 47% of participants scored above hepatitis B virus (HBV) and hepatitis B vaccination mean knowledge scores. Female sex (AOR 2.28, 95% CI 1.15-4.52, p<0.05), perceiving there to be an occupational risk of exposure to HBV (AOR 2.86, 95% CI 1.06-7.70, p<0.001), and increasing hepatitis B vaccination knowledge (AOR 2.68, 95% CI 1.83-3.92, p<0.001) were

For peer review only - http://bmjopen.bmj.com/site/about/guidelines.xhtml

independent predictors of full-dose hepatitis B vaccination in FRSC members, Kaduna Sector Command.

Conclusions: Hepatitis B vaccination coverage and knowledge are poor among FRSC members, Kaduna Sector Command. Institutionalizing free hepatitis B vaccination could improve uptake among FRSC members. Educational intervention, geared towards improving FRSC members' knowledge of hepatitis B vaccination and perception of risk of occupational exposure to HBV, is recommended for these vulnerable public safety workers. Such enlightenment could be a cheap and easy way of improving hepatitis B vaccination coverage in the study population.

Keywords: infection control; hepatitis B virus; vaccination coverage; public safety workers

ARTICLE SUMMARY

Strengths and Limitations of this Study

- This is the first study to estimate hepatitis B vaccination coverage of public safety workers such as the Federal Road Safety Corps in Nigeria despite these workers being occupationally exposed to hepatitis B virus.
- The participating Unit Commands were randomly selected and the study had a high response rate hence minimizing selection bias and improving the generalizability of the research findings.
- Retrospective studies are prone to recall bias; this was mitigated in this study by omitting inconsistent data suggestive of guessing at the analysis stage.
- Researcher bias was mitigated by the use of a pre-validated data collection instrument and by pre-determining analytical strategies before data collection while confounding was minimized through multivariate analysis.

For peer review only

For peer review only - http://bmjopen.bmj.com/site/about/guidelines.xhtml

- Missing data made sample size in multivariate analysis less than the pre-research estimate, though the proportion analyzed constituted a good representation of the entire study population.

INTRODUCTION

Hepatitis B virus (HBV) is a highly infectious blood-borne pathogen usually transmitted via percutaneous or mucosal exposure to infected blood and body fluids.[1] It is notorious for its chronic carrier deadly sequel of liver cirrhosis and hepatocellular carcinoma.[2] HBV infection affects about one third of the world's population with over 350 million persons being chronic carriers.[3] This results in >2 million deaths from chronic liver diseases annually.[4] Sub-Saharan Africa and East Asia have the highest HBV prevalence with about 5-10% of the entire adult population having chronic infections.[1]

Percutaneous exposures to HBV occur in adulthood either accidentally or through unsafe practices.[3] Transmissions via needle-stick and sharps injuries are frequent occurrences among health practitioners and vulnerable public safety workers (PSWs).[5-7] PSWs' occupational risk of HBV infection depends on their level of blood-skin exposure.[8-10] Woodruff et al. estimated 1.9 times (95% confidence interval (CI) 1.1-3.3) increased risk of infection with HBV in PSWs with blood-skin exposure than in their counterparts without such exposure.[10] The risk of transmission from infected blood is said to be 100 times more for HBV than for HIV in non-immune individuals.[2]

Prevention of new HBV infections in adulthood is a recognized global public health priority.[3] Hepatitis B vaccination (HBVc) is the most effective way of controlling HBV infection.[3] The World Health Organization (WHO) prescribes universal HBVc of healthcare workers (HCWs)

For peer review only - http://bmjopen.bmj.com/site/about/guidelines.xhtml

and PSWs with frequent exposure to blood.[11] A standard three-dose vaccine regimen, with the second and third doses given one month and six months apart from the initial dose respectively, is very effective in conferring immunity against HBV.[12] In healthy vaccinated adults, immunologic memory against HBV is retained for ≥20 years.[12]

Nigeria is hyperendemic for HBV, with a chronic carrier prevalence rate of up to 39%.[4,6,13] This prevalence is >4 times that noted in black South Africans (9.6%).[14] This status poses a great risk of occupational exposure to HBV for HCWs and PSWs with regular blood-skin contact, though this risk is yet to be estimated in any Nigerian study.

The Federal Road Safety Corps (FRSC) was established by the Federal Government of Nigeria in 1988 due to the high rate of road traffic crashes (RTCs) in the country.[15] Included in the road safety functions of FRSC are the rescue and emergency care of RTC victims which brings them in regular contact with blood.[15] The objectives of this study were to estimate HBVc coverage, and knowledge and socio-demographic determinants of full-dose uptake in FRSC members in Kaduna State, Nigeria, in order to inform relevant targeted vaccination policies.

METHODS

Study Design: A quantitative cross-sectional survey of FRSC members, KSC, Nigeria.

Setting and Target Population: Kaduna State is the third most populous State in Nigeria and has 3 senatorial zones with 23 local government areas (LGAs).

The FRSC is divided into 12 Zonal Commands; each Zonal Command has Sector Commands under it with each Sector Command being sub-divided into Unit Commands.[16] There are currently 204 Unit Commands in Nigeria.[16] The first 11 Unit Commands are located in the

For peer review only

KSC with the KSC headquarters making them 12 and these cover the entire 23 LGAs in Kaduna State (Table 1).

Table 1: Location, Coverage and Staff Distribution of Unit Commands (UCs) of FRSC, Kaduna Sector Command, Nigeria, June-July, 2015.

**Commands**	**Designation**	**Staff Strength**			**Number of LGAs Covered**	**Location (LGA)**
		Cadre				
		Officer	Marshal	Total		
Kaduna Sector Command (KSC) Headquarters	RS1.1	46	118	164	2	Kaduna North
Kafanchan UC	RS1.11	15	44	59	4	Jama'a
Birnin Gwari UC	RS1.12	17	35	52	1	Birnin Gwari
Zaria UC	RS1.13	24	66	90	5	Sabon Gari
Saminaka UC	RS1.14	10	36	46	1	Lere
Sabon Tasha UC	RS1.15	16	52	68	1	Chikun
Kakau UC	RS1.16	18	66	84	2	Chikun
Birnin Yero UC	RS1.17	15	44	59	1	Igabi
Gwantu UC	RS1.18	8	31	39	2	Sanga
Katari UC	RS1.19	19	37	56	1	Kachia
Kachia UC	RS1.110	10	26	36	2	Kachia
Tashan Yari UC	RS1.111	10	26	36	1	Makarfi
	**Total**	**208**	**580**	**789**	**23**	

KSC is one of the four Sector Commands that make up the RS1 Zonal Command whose headquarters is in Kaduna. There were 789 FRSC members in KSC at the time of this study. Two major cadres exist in FRSC: Officer and Marshal, though the latter is sub-divided into Marshal Inspectorate and Road Marshal Assistant. 26% (208/789) of FRSC members, KSC are

For peer review only - http://bmjopen.bmj.com/site/about/guidelines.xhtml

Officers while 74% (580/789) are Marshals. The study was carried out in six randomly selected Unit Commands: KSC headquarters, Saminaka, Kakau, Gwantu, Katari, and Kachia.

Inclusion and Exclusion Criteria: Only FRSC members in KSC aged 18 years and above with ≥6 months of service were included in the study. This ensured that only adults long enough in service to be made aware of the risk of HBV were surveyed. FRSC members working in the RS1 Zonal Command headquarters were excluded from the study as the Zonal Command includes other States beyond the study scope.

Sample Size: This was estimated using the formula for cross-sectional surveys: $n = 1.96^2 \times p(1-p)/d^2$, where n is the required sample size, p is prevalence estimate of HBVc in previous studies, and d is precision or acceptable error margin (5%).[17] Ogoina et al.'s prevalence estimate of 36.2% in a survey of 290 HCWs in Nigeria[6] was used as proxy since there is no existing study on PSWs in Nigeria. Anticipating a lower prevalence rate among non-HCWs with expectedly lower level of awareness of HBVc, 30% prevalence was assumed. ($N = 1.96^2 \times 0.3(1-0.3)/0.5^2 = 323$). Using 24% as anticipated non-response rate (q),[6] a final sample size of 425 was estimated using the formula: $N_f = N_s/1-q$, where N_f is the final sample size and N_s the initial sample size.[18]

Sampling: The sampling frame was a list of the 12 Unit Commands from the KSC headquarters. Each Unit Command was considered a cluster. Clusters were randomly selected using a computer-generated set of random numbers until sample size was achieved. This simple random selection of clusters was to ensure representativeness of selected Unit Commands.[19] Six Unit Commands were selected for the study. All FRSC members in the selected Unit Commands were targeted for questionnaire distribution.

For peer review only - http://bmjopen.bmj.com/site/about/guidelines.xhtml

Data Collection: Unit Commands of FRSC have compulsory weekly meetings. Permission was obtained for collection of data at these meetings. Data collection took place between 17th June and 22nd July, 2015. Participant information sheet (PIS) was reviewed with the staff with emphasis on voluntary participation, anonymity and confidentiality of collected data. Inclusion criteria and implied consent were further explained. Completion of questionnaire was considered consent to participate. Participants were asked to seal completed questionnaires in the given envelopes and drop them in a common collection box provided by the researcher. This was to ensure anonymity. Those not willing to participate were asked to drop the sealed uncompleted questionnaires in the box alongside participants. Non-responders were therefore not identified during data collection. Two Unit Commands (KSC Headquarters & Kakau) were re-visited in subsequent meetings due to poor initial attendance. Routine attendance lists taken by the Unit Commands at the initial meetings were used to prevent re-participation of previous participants.

Instruments: Due to paucity of studies on the research topic, accessing a pre-validated questionnaire for the study was difficult. After an extensive literature search, only Al-Hussami's "Hepatitis B Vaccine Knowledge and Acceptance" questionnaire could be found.[20] This questionnaire has been used for HCWs in United States. It was validated in two pilot studies with testing for inter-reliability but the test statistic was not reported.[20] There were 44 multiple choice questions including some open-ended ones. A structured anonymous self-administered questionnaire was adapted from this questionnaire for the present study (appendix A). Only questions relevant to the research questions were selected. Questions were simplified to suit the literacy status of the study population. The adapted study questionnaire contained 17 questions that elicited information on demographics (sex, age, duration of service, cadre and rank), HBV knowledge and perception of risk of exposure, and HBVc knowledge and status. Though rank

For peer review only - http://bmjopen.bmj.com/site/about/guidelines.xhtml

was obtained, this was not included in analysis since it mirrors cadre. The questionnaire was pilot-tested on FRSC members in RS1 Zonal Command headquarters.

Statistical Analysis: Table 2 describes the variables in the study.

Table 2: Description of Variables in the Study, FRSC, KSC, Nigeria, June-July, 2015.

Variable	Description	Type of Data
Independent Variables		
Sex	This was the gender of study participants categorized as either male or female	Nominal
Age	This variable ascertained the age of participants on their last birthday. It was categorized to enhance anonymity from 18 years which is the age definition of commencement of adulthood to ≥50 years which marks the age before retirement from Nigerian Civil Service at 60 years. The categories included: 18-29 years; 30-39 years; 40-49 years; ≥50 years	Ordinal
Duration of Service	This variable elicited how long a respondent had been in service with the Federal Road Safety Corps. It was categorized into: 6 months-2 years (probation period in civil service); 3- 10 years; 11 years to 19 years; and ≥20 years (close to retirement by service year at 35 years).	Ordinal
Cadre	This ascertained the official class of participant based on position and seniority in office. There were two major categories: Officers and Marshals with the latter sub-categorized into Marshal Inspectorate and Field Marshal Assistant in a descending order. It also signified educational qualification order with the least educated being the Field Marshal Assistant.	Nominal/Ordinal
Risk Perception	This ascertained the level of perception of occupational risk of exposure to HBV by respondents. It was initially categorized into: No risk of exposure, low risk of exposure, moderate risk of exposure, high risk of exposure, and I don't know. This was later	Nominal/Ordinal

For peer review only

Table 2: Description of Variables in the Study, FRSC, KSC, Nigeria, June-July, 2015.

Variable	Description	Type of Data
	dichotomized for further analysis by merging the 'I don't know' group with the 'no risk' group to form a 'no risk perceived' category with the rest forming the 'risk perceived' category.	
HBV Knowledge Score	This variable sought to estimate the level of knowledge of basic information on HBV. It includes questions on HBV awareness, seriousness compared to HIV, and route of transmission. For each participant, the number of questions answered correctly was noted as the score (see scoring table in appendix B).	Continuous
HBVc Knowledge Score	This measured the level of basic knowledge of hepatitis B vaccination among participants. It comprised questions on HBVc awareness, effectiveness, recommended full dosage and duration of protection from full-dose vaccination. For each participant, the number of questions answered correctly was noted as the score (appendix B).	Continuous
Dependent Variable		
Hepatitis B Vaccination (HBVc) Status	Information was elicited on whether participant had ever received HBVc and the number of doses received. Descriptive analysis was done using these data. Dichotomization of data was also done for logistic regression analysis. Since only those with ≥3 doses of HBVc uptake are considered fully protected,[2] those with ≥3 doses were labeled 'vaccinated' and the rest 'not vaccinated'. This was noted as the HBVc status of each participant.	Nominal

All analyses were conducted using statistical package for social sciences version 21. Descriptive analysis ascertained frequencies and distributions of data. Histograms showed both HBV knowledge and HBVc knowledge scores to be normally distributed, hence their mean and standard deviations (SD) were calculated as was the percentage of participants scoring above the

For peer review only

For peer review only - http://bmjopen.bmj.com/site/about/guidelines.xhtml

mean scores. Since the outcome variable (HBVc status) was binary, logistic regression analysis was used in testing for associations with the independent variables (sex, age, duration of service, cadre, risk perception, and HBV and HBVc knowledge scores).[21] To mitigate confounding, univariate analyses were first carried out and the variables identified as significantly associated ($p<0.05$) with HBVc status were included in the multivariate analysis for independent predictors of full-dose HBVc uptake.[21] Adjusted odds ratios (AOR) with 95% confidence interval (CI) for each variable was computed and significance level set at $p<0.05$. Missing data on each variable were excluded in the analysis of the variable.

RESULTS

354 questionnaires were distributed in the six Unit Commands sampled from FRSC, KSC. 6 questionnaires were discarded for having missing data on up to 3 of the independent variables or on the dependent variable and 2 or more independent variables. 7 questionnaires were submitted blank. 341 completed questionnaires were included for analysis, giving a response rate of 96.3%. Appendix C shows percentage of missing data for each of the 14 questions analyzed. Missing data were most frequent on the question on recommended dose of vaccine (9.7%; 33/341) followed by that on the duration of protection from full-dose HBVc (8.5%; 29/341). All participants provided data on cadre. Most respondents were males; aged 30-39 years; had worked between 3-10 years with FRSC; and were of Marshal Cadre (table 3).

For peer review only - http://bmjopen.bmj.com/site/about/guidelines.xhtml

Table 3: Socio-Demographic Characteristics of Study Sample of FRSC Members, KSC, Nigeria, June-July, 2015.

Variable	Frequency	Percentage
Sex (n=327)		
Male	260	79.5
Female	67	20.5
Age (n=338)		
18-29 years	64	18.9
30-39 years	167	49.4
40-49 years	87	25.7
≥50 years	20	5.9
Duration of Service (n=339)		
6 months-2 years	36	10.6
3-10 years	188	55.5
11-19 years	87	25.7
≥20 years	28	8.3
Cadre (n=341)		
Officer	96	28.2
Marshal	245	71.8
-Marshal Inspectorate	111	32.6
- Field Marshal Assistant	134	39.3

HBV Knowledge: The mean total number of correct answers to HBV knowledge questions was 3.0 out of 6.0 (SD 1.5). 46% (157/341) of participants scored above the mean. The proportion of correct answers to HBV knowledge questions ranged from 2.1% (7/337) on route of transmission of HBV to 93.2% (317/340) on having ever heard of HBV. 22.6% (76/337) of respondents answered 'I don't know' to the question pertaining to the route of transmission of HBV and this response was the most frequent. Merely 2.1% (7/337) correctly identified contact with infected blood and blood-contaminated body fluid as routes of transmission of HBV. Only 4 participants (1.2%; n=341) answered all 6 HBV knowledge questions correctly while 16 (4.7%, n=341)

For peer review only - http://bmjopen.bmj.com/site/about/guidelines.xhtml

answered none correctly. HBV infection was perceived as more serious than HIV by most respondents (56.7%; 190/335) while about 3.0% (10/335) felt it was less serious than HIV. 20.6% (69/335) claimed no knowledge of the seriousness of HBV compared to HIV while 19.7% (66/335) ascribed equal severity to the two.

HBVc Knowledge: The mean number of correct answers to HBVc questions was 2.0 out of 4.0 (SD 1.1). 42.2% (144/341) of participants had scores higher than the mean score. All four questions on HBVc were answered correctly by only 4.1% (14/341) of participants while no correct answer was given by 11.7% (40/341). Rate of correctness ranged from 6.1% (19/312) on question on duration of protection from full-dose HBVc to 86.6% (291/336) on having ever heard of HBVc. Most respondents (62.9%; 210/334) described HBVc as very effective. While 6.9% (23/334) rated it slightly effective, 2.7% (9/334) felt it was not effective at all. 27.5% (92/334) of respondents indicated not knowing its effectiveness. 54.9% (169/308) of responding participants correctly identified recommended full HBVc dose as ≥3 doses while 1.6% (5/308) and 3.9% (12/308) thought it was 1 dose and 2 doses respectively. Up to 39.6% (122/308) indicated not knowing the recommended full dose of HBVc.

Perception of Risk of Occupational Exposure to HBV: While most respondents (55.3%; 188/340) rated themselves at high risk of occupational exposure to HBV, 22.4% (76/340) did not know their risk status. 5.3% (18/340) of respondents considered themselves at no risk of exposure to HBV while 5.9% (20/340) and 11.2% (38/340) rated themselves at low and moderate risks of exposure respectively. After dichotomizing this variable into 'no risk perceived' and 'risk perceived' categories, 72.4% (246/340) had some level of risk perception while 27.6% (94/340) had no risk perception for HBV.

For peer review only - http://bmjopen.bmj.com/site/about/guidelines.xhtml

HBVc Coverage: Of the 341 participants, 6 did not provide data on their HBVc status. 10 others answered ‘yes’ to having ever received HBVc but omitted the number of doses received and were therefore inputted as missing data. 325 respondents (95.3%) were included in the sub-analysis. 60.9% (198/325) of the respondents affirmed having ever received ≥1 dose of HBVc. 50.0% of these (99/198) had received ≥3 doses resulting in full-dose coverage of 30.5% (99/325) among the respondents. 39.1% (127/325) of respondents had never received HBVc. Together with the 99 participants with <3 doses, 69.5% (226/325) were classified ‘not vaccinated’ while 30.5% (99/325) were labeled ‘vaccinated’.

Logistic Regression Analyses: All the variables were significantly associated with HBVc on univariate analyses (table 4) and were included in the multivariate analysis for independent predictors of full-dose HBVc uptake (table 5). Being female was associated with about twice the likelihood of having received full-dose HBVc (table 5). When risk perception was analyzed as a dichotomous variable (‘no risk perceived’ versus ‘risk perceived’), those with any level of risk perception for occupational exposure to HBV were about 3 times more likely to have received full-dose HBVc than those without risk perception for HBV (table 5). Though the odds of being fully vaccinated increased with duration of service, this was not statistically significant. While HBV knowledge was not a significant predictor of full-dose HBVc, knowledge of HBVc was significantly associated with full-dose HBVc with each unit increase in number of correct answers being associated with up to three times increased likelihood of being fully vaccinated (table 5).

For peer review only - http://bmjopen.bmj.com/site/about/guidelines.xhtml

Table 4: Univariate Binary Logistic Regression Analyses Showing Unadjusted Odds Ratios of the Association between Independent Variables and Full-Dose Hepatitis B Vaccination Uptake among FRSC Members, KSC, Nigeria, June-July, 2015

Independent Variable	Sample Size	Odds Ratio	95% CI (p-value)
Sex	313		
Male		1	
Female		2.66	1.51-4.70 (0.001)
Age	323		
18-29 years		1	
30-39 years		2.08	0.97-4.44 (0.059)
40-49 years		3.30	1.47-7.40 (0.004)
≥50 years		5.67	1.84-17.50 (0.003)
Duration of service	323		
6 months-2 years		1	
3-10 years		5.69	1.31-24.72 (0.020)
11-19 years		9.48	2.12-42.35 (0.003)
≥20 years		13.39	2.61-68.56 (0.002)
Cadre	325		
Officer		1	
Marshal Inspectorate		1.69	0.92-3.09 (0.091)
Road Marshal Assistant		2.10	1.18-3.74 (0.012)
Risk Perception for Occupational Exposure to HBV	324		
I don't know		1	
No risk		3.92	0.78-19.63 (0.096)
Low risk		11.33	2.94-43.63 (<0.001)
Moderate risk		9.61	2.85-32.43 (<0.001)
High risk		11.07	3.87-31.70 (<0.001)
Risk perception for Occupational Exposure to HBV	324		
No risk perceived		1	
Risk perceived		7.39	3.27-16.71 (<0.001)
HBV Knowledge Score	325	1.37	1.15-1.62 (<0.001)
HBVc Knowledge Score	325	2.97	2.16-4.08 (<0.001)

For peer review only

For peer review only - http://bmjopen.bmj.com/site/about/guidelines.xhtml

Table 5: Multivariate Binary Logistic Regression Analysis for Independent Predictors of Full-Dose Hepatitis B Vaccination Uptake among Members of FRSC, KSC, Nigeria, June-July, 2015.

Independent Variable	Adjusted Odds Ratio n=309	95% CI (p-value)
Sex		
Male	1	
Female	2.28	1.15-4.52 (0.019)
Age		
18-29 years	1	
30-39 years	1.40	0.47-4.18 (0.542)
40-49 years	0.99	0.28-3.55 (0.987)
≥50 years	1.08	0.20-5.76 (0.931)
Duration of service		
6 months-2 years	1	
3-10 years	2.12	0.39-11.41 (0.384)
11-19 years	2.73	0.45-16.59 (0.276)
≥20 years	5.25	0.68-40.47 (0.112)
Cadre		
Officer	1	
Marshal Inspectorate	1.60	0.77-3.33 (0.208)
Road Marshal Assistant	0.87	0.41-1.85 (0.720)
Risk Perception for Occupational Exposure to HBV		
I don't know	1	
No risk	2.93	0.47-18.41 (0.251)
Low risk	7.12	1.47-34.47 (0.015)
Moderate risk	4.50	1.03-19.63 (0.045)
High risk	3.90	1.08-14.09 (0.038)
Risk perception for Occupational Exposure to HBV		
No risk perceived	1	
Risk perceived	2.86	1.06-7.70 (<0.001)
HBV Knowledge Score	1.03	0.80-1.31 (0.843)
HBVc Knowledge Score	2.68	1.83-3.92 (<0.001)

226

227 In summary, full-dose HBVc was 30.5% while any dose coverage was 60.9%. Female sex,
228 perceiving their occupation as conveying a risk of HBV, and increasing HBVc knowledge were

For peer review only - http://bmjopen.bmj.com/site/about/guidelines.xhtml

significant independent predictors of full-dose HBVc uptake among members of FRSC in Kaduna State, Nigeria.

DISCUSSION

Like all other studies on HCWs in Nigeria,[13,23-25] this study has further demonstrated high HBVc initiation rate (60.9%) with low completion rate (30.5%). This completion rate implies that only 30.5% of members of the FRSC, KSC were adequately protected against HBV infection.[2] This means that almost 70% of these rescue workers perform their duties without adequate protection from HBV. This also places the accident victims whom they seek to rescue at risk of infection with HBV from infected FRSC members. This could lead to an unbroken cycle of infectivity, morbidity and mortality from HBV in a nation still struggling with the HBV scourge. Poor uptake of HBVc among those at occupational risk of exposure to HBV in Nigeria is a common observation across studies.[13,22-27] Non-existence of a universal HBVc policy in the country for HCWs and vulnerable PSWs could be contributory to poor vaccine uptake. HBVc is sourced individually by workers except for sporadic free immunization programmes in some institutions. Lee et al. demonstrated up to 78% HBVc coverage among a subset of PSWs in United States which has institutionalized HBVc policy for HCWs and vulnerable PSWs.[28] The Centers for Disease Control and Prevention (CDC) recommends routine immunization of HCWs against HBV.[2] Occupational Safety and Health Administration (OSHA) designated police and firefighters as HCWs considering their often adopted role of emergency medical service providers.[7] FRSC members are exposed to blood and sharps injuries from accident scenes. Though there are no statistical estimates from previous studies to quantify their occupational exposure levels, their job descriptions and high prevalence of RTCs in Nigeria presumably place them at high risk of exposure to HBV in this hyperendemic setting.[15,29] In a nation with 39%

For peer review only - http://bmjopen.bmj.com/site/about/guidelines.xhtml

prevalence for chronic HBV,[4] HBVc coverage of 30.5% for FRSC members, KSC is low. OSHA includes workers in public safety institutions among personnel to receive mandatory free HBVc provided at employer's cost as contained in the "Bloodborne Pathogens Standard".[30,31] From this study, it is recommended that the Federal Government of Nigeria, through the Federal Ministry of Health, provide free HBVc to all FRSC staff in KSC who are not yet fully vaccinated, and enact a policy to institutionalize free mandatory HBVc for all unimmunized trainees at the initial basic training programme for newly recruited staff. This will ensure adequate HBVc coverage of FRSC staff before exposure to rescue operations.

Among FRSC members, females are 2.28 times more likely to be fully vaccinated against HBV compared to their male counterparts. Osazuwa-Peters et al. observed a similar but insignificant female preponderance in HBVc among dental professionals in Edo State, Nigeria.[32] Contrarily, Adekanle et al., in their survey of HCWs in Ile-Ife, Nigeria, observed 1.8 times increased chances of males receiving HBVc compared to females,[26] although this finding may be due to males in the study being mainly doctors who had the advantage of professional knowledge of HBVc. It is possible that, in the present study, females could be exposed to HBVc knowledge during antenatal hospital visits and while taking their children for immunization.

More than 50% of respondents perceived themselves at high risk of occupational exposure to HBV. Disturbingly though, close to a quarter of the study participants claimed no knowledge of their risk status. Together with those who perceived themselves to be at no risk, 27.6% of FRSC members had no risk perception for occupational exposure to HBV while 72.4% perceived themselves at risk. This falls within the range of 30% to 78% risk perception observed in studies among HCWs in Nigeria.[13,27] While the rate of risk perception was high, having approximately 28% with no risk perception for HBV is quite disconcerting from a public health

For peer review only - http://bmjopen.bmj.com/site/about/guidelines.xhtml

perspective considering the blood-skin exposure that rescue of accident victims could entail. Perception of threat of a disease provides cue for action in favour of a health-promoting behaviour.[33] This was demonstrated in this study as those who perceived themselves at risk of occupational exposure to HBV were three times more likely to be vaccinated than those with no risk perception. All risk categories had higher odds of vaccination compared to those without risk perception. It is therefore important that FRSC members understand the risk of exposure to HBV (even if they feel it is low) as this appears to increase their likelihood of getting vaccinated.

Knowledge of HBV and HBVc among study participants was poor. Less than 47% of participants scored above the mean knowledge scores for HBV and HBVc. Knowledge was poorest for the route of transmission of HBV and duration of protection from full-dose HBVc. Not knowing the route of transmission of HBV means that FRSC members might not take adequate precautions during rescue operations nor adopt preventive measures against the virus. It could also lead to stigmatization of FRSC members already infected with HBV due to wrong assumption of infectivity through casual contact with their sweat and saliva. HBV knowledge score was however not an independent predictor of HBVc in the study. This conflicts with Adekanle et al.'s observation of twice increased likelihood of complete HBVc among those with good knowledge of HBV in their survey of HCWs in Ile Ife, Nigeria.[26] Their study though did not elicit information on HBVc knowledge as was done in this study. HBVc knowledge could have confounded the demonstrated association between HBV knowledge and HBVc uptake.

Only 6.1% of participants knew that full-dose HBVc gives protection for ≥20 years. This was despite the description of HBVc as very effective by 62.9% of participants. Knowing that receiving ≥3 doses of the vaccine can give one lifetime protection from HBV could incentivize full-dose uptake among FRSC members. HBVc knowledge was the most significant and precise

For peer review only - http://bmjopen.bmj.com/site/about/guidelines.xhtml

independent predictor of full-dose HBVc in this study. This contradicts Ogoina et al.'s finding of no significant association between knowledge of HBVc and full-dose vaccination among HCWs in two tertiary hospitals in Nigeria.[6] However, they did not ascertain knowledge of vaccine effectiveness and duration of protection from full-dose vaccination.

Educational programme towards improvement of HBV and HBVc knowledge, and risk perception among FRSC members is a recognized relevant public health intervention from this study. The programme can be included in the schedules of the already existing compulsory weekly in-house training/manpower development of staff and in the routine basic training programme for new staff. Such enlightenment would be a cheap and easy intervention to improve HBVc uptake. Existing evidence on the positive impact of educational intervention on vaccine uptake is however weak.[34] The educational intervention should therefore be rigorously evaluated to ascertain its impact on HBVc uptake in FRSC members.

Study Strengths and Limitations:

This was a descriptive cross-sectional survey which limits its suitability for demonstrating temporal relationships between explanatory and outcome variables.[35] It nevertheless shows independent associations useful in understanding predictors of full-dose HBVc in this study population so as to inform relevant public health interventions. Recall bias is another limitation of this retrospective study design as participants may not have remembered accurately their vaccination history, which could have introduced information bias.[36] However, this was addressed at the analysis stage by omitting inconsistent data suggestive of guessing. Simple random sampling using the staff register would have been most ideal in selecting a representative sample with minimal selection bias;[21] this was not feasible considering the disproportionate

For peer review only - http://bmjopen.bmj.com/site/about/guidelines.xhtml

distribution of some of the socio-demographic variables like sex and cadre in the study population. Also, the response rate of 96.3% was impressive and minimizes selection bias wherein non-participants could differ significantly from participants in the study variables.[37] This enhances the generalizability of research findings by improving the external and internal validity of the study.[37] The presence of Unit Commanders and other senior members of staff at the meetings during data collection and their participation in the research could have contributed to the high response rate. Subordinates who ordinarily may not have wished to participate could have felt a psychological obligation to participate with their bosses. This power influence was minimized by the use of PIS which emphasized voluntary participation, and by anonymous data collection procedures. Quantitative studies are prone to researcher bias.[21] This was minimized by the use of a pre-validated questionnaire and by pre-determining analytical strategies before data collection. Possible exchange of information among participants could have introduced information bias. This was mitigated by the presence of the researcher during data collection with prior emphasis on non-communication between participants. Though the study's questionnaire was adapted and pilot-tested to reflect the study context, it was not tested for inter-rater reliability and validity within the study population. Missing data reduced the sample size for the multivariate analysis from 341 to 309. This sample size was less than the pre-study estimate (323) and could lack sufficient power to detect significant associations, hence predisposing to type II error.[21] It however constitutes a randomized 39% (309/789) of the study population, which is a good representation.[38] Confounding, a known menace in observational studies, was minimized at the analytical stage through multivariate logistic regression.[21] The research estimates on the association of varied levels of risk perception with

For peer review only - http://bmjopen.bmj.com/site/about/guidelines.xhtml

full-dose HBVc were not precise, having very wide 95% CIs. This could be due to random errors in the sample.[36] A larger sample size in future studies could yield more precise estimates.

CONCLUSION

Persons with chronic HBV are at 15%-25% risk of premature mortality from the sequels of liver cirrhosis and hepatocellular carcinoma.[39]. Controlling HBV transmission is an important public health issue internationally and in Nigeria where the virus is hyperendemic. HBV infection is a preventable disease and prevention is best achieved with HBVc.[3] FRSC members come in regular contact with blood and are at risk of contracting the virus. HBVc coverage among FRSC members in Kaduna State, Nigeria is low (30.5%). Knowledge of HBV and HBVc is poor in this study population. Female sex, perceiving there to be an occupational risk of exposure to HBV, and increasing HBVc knowledge are independent predictors of HBVc uptake among FRSC members, KSC. Institutionalized free HBVc is recommended for FRSC staff. Educational intervention aimed at creating proper awareness of the occupational risk of HBV and the importance of HBVc, is required to improve HBVc coverage among this vulnerable group of PSWs. Recommended future studies include: a qualitative study to ascertain FRSC members' perception of HBVc and subjective reasons for non-uptake of the vaccine; a sero-prevalence study to determine the actual immune status of FRSC members in KSC and estimate the prevalence of HBV in this study group for appropriate intervention; and validation of questionnaire in the Nigerian context with pretesting and retesting for reliability.

ACKNOWLEDGMENTS: University of Liverpool is acknowledged for providing the platform for this research. We thank the Commonwealth Scholarship Commission for paying the tuition fee for the MPH study of the corresponding author thereby enabling this dissertation

For peer review only - http://bmjopen.bmj.com/site/about/guidelines.xhtml

research. We appreciate the staff and management of the Federal Road Safety Corps, Kaduna Sector Command for their cooperation and participation in this study.

REFERENCES

1. World Health Organization (WHO). Hepatitis B *WHO* 2015. http://www.who.int/mediacentre/factsheets/fs204/en/ (Accessed: 23rd July 2015).
2. Centers for Disease Control and Prevention (CDC). Immunization of Health-Care Personnel: Recommendations of the Advisory Committee on Immunization Practices (ACIP). *Morbidity and Mortality Weekly Report (MMWR): Recommendations and Reports* 2011;60(RR07):1-45. http://www.cdc.gov/mmwr/preview/mmwrhtml/rr6007a1.htm (Accessed: 12th July 2015).
3. Lavanchy D. Chronic Viral Hepatitis as a Public Health Issue in the World. *Best Practice & Research Clinical Gastroenterology* 2008;22(6):991-1008.
4. Emechebe GO, Emodi IJ, Ikefuna AN, et al. Hepatitis B Virus Infection in Nigeria- A Review. *Niger Med J* 2009;50:18-22.
5. Fekadu GA, Azage M. Hepatitis B Vaccine Knowledge and Vaccination Status among Health Care Workers of Bahir Dar City Administration, Northwest Ethiopia: A Cross-sectional Study. *BMC Infectious diseases* 2015;15:475-87.
6. Ogoina D, Pondei K, Adetunji B, et al. Prevalence of Hepatitis B Vaccination among Health Care Workers in Nigeria in 2011-12. *Int J Occup Environ Med* 2014;5:51-56.
7. Occupational Safety and Health Administration (OSHA). Standard Interpretations. In: Bloodborne Pathogens Standard. USA:OSHA 2004;29 CFR 1910.1030(f)(1).

For peer review only - http://bmjopen.bmj.com/site/about/guidelines.xhtml

https://www.osha.gov/pls/oshaweb/owadisp.show_document?p_table=INTERPRETATIONS&p_id=24010 (Accessed: 11July 2015).

8. Boal WL, Hales T, Ross CS. Blood-Borne Pathogens among Firefighters and Emergency Medical Technicians. *Prehosp Emerg Care* 2005;9:236-47.

9. Rischitelli G, Harris J, McCauley L, et al. The Risk of Acquiring Hepatitis B or C among Public Safety Workers- A Systematic Review. *Am J Prev Med* 2001;20:299-306.

10. Woodruff BA, Moyer LA, O'Rourke KM, Margolis HS. Blood Exposure and the Risk of Hepatitis B Virus Infection in Firefighters. *J Occup Med* 1993;35:1048-54.

11. World Health Organization (WHO). Table 1: Summary of WHO Position Papers- Recommendations for Routine Immunization. *Immunization, Vaccines and Biologicals* 2014. http://www.who.int/immunization/policy/immunization_tables/en/ (Accessed: 20 February 2015).

12. Centers for Disease Control and Prevention (CDC). Hepatitis B FAQs for Health Professionals. *Viral Hepatitis – Hepatitis B Information* 2015. http://www.cdc.gov/hepatitis/hbv/hbvfaq.htm#treatment (Accessed: 6th June 2015).

13. Ibekwe RC, Ibeziako N. Hepatitis B Vaccination Status among Health Workers in Enugu. *Nigerian Journal of Clinical Practice* 2006;9:7-10.

14. Burnett RJ, Francois G, Mphahlele MJ, et al. Hepatitis B Vaccination Coverage in Healthcare Workers in Gauteng Province, South Africa. *Vaccine* 2011:29:4293-97.

15. Chidoka O. The Lead Agency Concept: Federal Road Safety Corps, Nigeria Experience. Paper presented at the African Road Safety Management Workshop; 2014; Freetown, Sierra Leone.

For peer review only

For peer review only - http://bmjopen.bmj.com/site/about/guidelines.xhtml

http://www.ssatp.org/sites/ssatp/files/pdfs/Events/2014/Presentation%20Nigeria.pdf (Accessed: 19 April 2015).

16. Federal Road Safety Corps (FRSC). Official Website. *Federal Government of Nigeria* 2015. http://frsc.gov.ng/ (Accessed: 22 February 2015).

17. Charan J, Biswas, T. How to Calculate Sample Size for Different Study Designs in Medical Research? *Indian J Psychol Med* 2013;35:121-6.

18. Whitley E, Ball J. Statistics Review 4: Sample Size Calculations. *Crit Care* 2002;6:335-41.

19. Hammer GP, Prel J-B, Blettner M. Avoiding Bias in Observational Studies. *Dtsch Arztebl Int* 2009;106:664-8.

20. Al-Hussami M. Knowledge and Acceptance of Hepatitis B Vaccine. *The Internet Journal of Healthcare Administration* 2001;2. https://ispub.com/IJHCA/2/1/5725 (Accessed: 20th April 2015).

21. Bruce N, Pope D, Stanistreet D. Quantitative Methods for Health Research: A practical Interactive Guide to Epidemiology and Statistics. Chichester,UK: Wiley 2008.

22. Azodo CC, Ehizele AO, Uche I, Erhabor P. Hepatitis-B Vaccination Status among Dental Surgeons in Benin City, Nigeria. *Ann Med Health Sci Res* 2012;2:24-8.

23. Fatusi AO, Fatusi OA, Esimai AO, et al. Acceptance of Hepatitis B Vaccine by Workers in a Nigerian Teaching Hospital. *East African Medical Journal* 2000;77:608-12.

24. Okwara EC, Enwere OO, Diwe CK, et al. Theatre and Laboratory Workers' Awareness of and Safety Practices against Hepatitis B and C Infection in a Suburban University Teaching Hospital in Nigeria. *Pan African Medical Journal* 2012;13.

For peer review only - http://bmjopen.bmj.com/site/about/guidelines.xhtml

25. Paul N, Peterside O. Hepatitis B Vaccination Rate among Medical Students at the University of Port Harcourt Teaching Hospital (UPTH). *World Journal of Vaccines* 2015;55:1-7. http://dx.doi.org/10.4236/wjv.2015.51001 (Accessed: 22 June 2015).

26. Adekanle O, Ndububa DA, Olowookere SA, et al. Knowledge of Hepatitis B Virus Infection, Immunization with Hepatitis B Vaccine, Risk Perception, and Challenges to Control Hepatitis among Hospital Workers in a Nigerian Tertiary Hospital. *Hepatitis Research and Treatment* 2015;2015:1-6 [Online]. Available from: http://dx.doi.org/10.1155/2015/439867 (Accessed: 23rd May 2015).

27. Adebamowo CA, Odukogbe AA, Ajuwon AJ. Knowledge, Attitude, and Practices Related to Hepatitis B Virus Infection among Nigerian Obstetricians and Midwives. *J Obstet Gynaecol* 1998;18:528-32.

28. Lee DJ, Carrillo L, Fleming L. Epidemiology of Hepatitis B Vaccine Acceptance among Urban Paramedics and Emergency Medical Technicians. *Am J Infect Control* 1997;25:421-3.

29. Labinjo M, Juillard C, Kobusingye OC, Hyder AA. The Burden of Road Traffic Injuries in Nigeria: Results of a Population-Based Survey. *Inj Prev* 2009;15:157-62.

30. Occupational Safety and Health Administration (OSHA). Bloodborne Pathogens Standard. USA:OSHA 2001;29 CFR 1910.1030. https://www.osha.gov/pls/oshaweb/owadisp.show_document?p_table=STANDARDS&p_id=10051 (Accessed: 11th July 2015).

31. Occupational Safety and Health Administration (OSHA). Hepatitis B Vaccination Protection. In: OSHA Fact Sheet 2011.

For peer review only - http://bmjopen.bmj.com/site/about/guidelines.xhtml

https://www.osha.gov/OshDoc/data_BloodborneFacts/bbfact05.pdf (Accessed: 11th July 2015).

32. Osazuwa-Peters N, Azodo CC, Ehizele AO, Obuekwe ON. Gender Differences in Characteristics, Occupational Exposure, and Infection Control Practices among Dental Professionals in Edo State, Nigeria. *South Afr J of Epidemiol Infect* 2012;27:61-5.

33. Adams A, Hall M, Fulghum J. Utilizing the Health Belief Model to Assess Vaccine Acceptance of Patients on Hemodialysis. *Nephrol Nurs J* 2014;41:393-406.

34. Dube E, Gagnon D, MacDonald NE, the SAGE Working Group on Vaccine Hesitancy. Strategies Intended to Address Vaccine Hesitancy: Review of Published Reviews. *Vaccine* 2015;33:4191-203.

35. Carlson MDA, Morrison RS. Study Design, Precision, and Validity in Observational Studies. *J Palliat Med* 2009;12:77-82.

36. Neugebauer R, Ng S. Differential Recall as a Source of Bias in Epidemiologic Research. *J Clin Epidemiol* 1990;43:1337-41.

37. Knottnerus JA, Tugwell P. Selection-Related Bias, An Ongoing Concern in Doing and Publishing Research. *J Clin Epidemiol* 2014;67:1057-8. http://www.jclinepi.com/article/S0895-4356(14)00332-1/pdf (Accessed: 15 September 2015).

38. Kadam P, Bhalerao, S. Sample Size Calculation. *Int J Ayurveda Res* 2010;1:55-57.

39. World Health Organization (WHO). Hepatitis B Vaccines: WHO Position Paper *Weekly Epidemiological Record* 2009;84:405-20. http://www.who.int.ezproxy.liv.ac.uk/wer/2009/wer8440.pdf (Accessed: 20 June 2015).

For peer review only

For peer review only - http://bmjopen.bmj.com/site/about/guidelines.xhtml

For peer review only

For peer review only - http://bmjopen.bmj.com/site/about/guidelines.xhtml

APPENDIX A: ADAPTED STUDY QUESTIONNAIRE

HEPATITIS B VACCINATION QUESTIONNAIRE

For Official Use Only

Researcher…………………………………… Questionnaire no: ☐

Date of Data Collection (DD/MM/YY)……………………….

IMPLIED CONSENT (Please read before completing questionnaire): Having gone through the research information contained in the participant information sheet, by completing this questionnaire you are consenting to participate in the study. If you do not wish to complete the questionnaire, please put the blank version into the envelope and I will collect it with all other questionnaires. Thank you.

Please only complete the questionnaire if you are aged 18 years and above and have at least 6 months of service with FRSC

Note: This questionnaire is anonymous; please do not write your name on it. Kindly give an answer to all the questions as it pertains to you and please answer as truthfully as you can.

Please check (✔) only the box that most correctly answers the question, making sure you make only one selection for each question except where otherwise indicated.

Section A: Demographic Questions

1. What is your sex?

☐ Male

☐ Female

For peer review only

2. What was your age on your last birthday?

- ☐ 18 to 29 years
- ☐ 30 to 39 years
- ☐ 40 to 49 years
- ☐ 50 years and above

Section B: Employment History

3. How long have you worked with Federal Road Safety Corps (FRSC)?

- ☐ 6 months to 2 years
- ☐ 3 years to 10 years
- ☐ 11 years to 19 years
- ☐ 20 years and above

4. What is your cadre?

- ☐ Officer
- ☐ Marshal Inspectorate
- ☐ Road Marshal Assistant

5. Please check (✔) the box below your rank

ACM	CC	DCC	ACC	CRC	SRC	RC	DRC	ARC

CI	DCI	ACI	PMI	SMI	MI-I	MI-II	MI-III	CRMA	DCRMA	SRMA	RMAI	RMAII	RMAIII

For peer review only

Section C: Perception of Risk of Exposure to Hepatitis B Virus

6. Have you ever heard about hepatitis B virus infection?

☐ Yes

☐ No

7. How serious do you think being infected with hepatitis B virus is compared to HIV?

☐ Less serious than HIV

☐ As serious as HIV

☐ More serious than HIV

☐ I don't know

8. How can someone be infected with hepatitis B virus? (please check (✔) all the correct boxes if your answer is more than one)

☐ Through contact with blood of an infected person

☐ Through contact with saliva of an infected person

☐ Through contact with sweat of an infected person

☐ Through contact with body fluid contaminated by blood of an infected person

☐ I don't know

For peer review only

9. How much do you think your work with FRSC exposes you to the risk of contracting hepatitis B virus infection?

- ☐ No risk of exposure
- ☐ Low risk of exposure
- ☐ Moderate risk of exposure
- ☐ High risk of exposure
- ☐ I don't know

Section D: Hepatitis B vaccination Knowledge and Status

10. Have you ever heard about hepatitis B vaccination?

- ☐ Yes
- ☐ No

11. How effective do you think hepatitis B vaccination is in protecting someone against hepatitis B virus infection?

- ☐ Not effective
- ☐ Slightly effective
- ☐ Very effective
- ☐ I don't know

12. Have you ever received hepatitis B vaccination?

- ☐ Yes
- ☐ No

If your answer to question 12 is 'No', answer question 13; if it is 'Yes', go to question 14

For peer review only

13. Why have you not received hepatitis B vaccination? (Please check (✔) all the correct boxes if your answer is more than one)

- ☐ I am not aware of hepatitis B vaccination
- ☐ I do not know where to go and receive it
- ☐ I don't have time
- ☐ It is expensive
- ☐ I don't see the need
- ☐ I am afraid of contracting the virus from the vaccine
- ☐ Others (please state)……………………………………………………………………………

14. If your answer to question 12 is 'Yes', how many doses of hepatitis B vaccine have you received?

- ☐ 1 dose
- ☐ 2 doses
- ☐ 3 doses
- ☐ More than 3 doses

15. When did you receive the last dose of hepatitis B vaccine?

- ☐ Less than 1 month ago
- ☐ 1 month to 3 months ago
- ☐ 4 months to 6 months ago
- ☐ More than 6 months ago

For peer review only

16. What do you think is the recommended full dose of hepatitis B vaccine?

- ☐ 1 dose
- ☐ 2 doses
- ☐ 3 or more doses
- ☐ I don't know

17. How long does a full dose of hepatitis B vaccine protect someone?

- ☐ Less than 1 year
- ☐ 1 year to 5 years
- ☐ 6 years to 10 years
- ☐ 11 years to 19 years
- ☐ 20 years or more
- ☐ I don't know

Thank you for your time!

For peer review only

APPENDIX B: Knowledge Scoring Table

Scoring Table: HBV (A) and HBVc (B) Knowledge Questions and Scoring Pattern

**A. Question**	**Options checked**	**Score**
Have you heard about hepatitis B virus infection? (**one option**)	Yes (✔)<br><br>No (✔)	1<br><br>0
How serious do you think being infected with hepatitis B virus is compared to HIV? (**one option**)	Less serious than HIV (✔)<br><br>As serious as HIV (✔)<br><br>More serious than HIV (✔)<br><br>I don't know (✔)	0<br><br>0<br><br>1<br><br>0
How can someone be infected with hepatitis B virus? (please check (✔) all the correct boxes if your answer is more than one)	Through contact with blood of an infected person (✔)<br><br>Through contact with saliva of an infected person (**blank**)*<br><br>Through contact with sweat of an infected person (**blank**)*<br><br>Through contact with body fluid contaminated by blood of an infected person (✔)<br><br>I don't know (✔)	1<br><br>1<br><br>1<br><br>1<br><br>0 for all options
Maximum HBV knowledge Score<br>Minimum HBV knowledge Score		6 of 6<br>0 of 6
**B. Question**	**Options**	**Score**
Have you ever heard about hepatitis B vaccination? (**one option**)	Yes (✔)<br><br>No (✔)	1<br><br>0
How effective do you think hepatitis B vaccination is in protecting someone against hepatitis B virus infection? (**one option**)	Not effective (✔)<br><br>Slightly effective (✔)<br><br>Very effective (✔)<br><br>I don't know (✔)	0<br><br>0<br><br>1<br><br>0
What do you think is the recommended full dose of hepatitis B vaccine? (**one option**)	1 dose (✔)<br><br>2 doses (✔)<br><br>3 or more doses (✔)<br><br>I don't know (✔)	0<br><br>0<br><br>1<br><br>0
How long does a full dose of hepatitis B vaccine protect	Less than 1 year (✔)	0

For peer review only

A. Question	Options checked	Score
someone? (**one option**)	1 year to 5 years (✔)	0
	6 years to 10 years (✔)	0
	11 years to 19 years (✔)	0
	20 years or more (✔)	1
	I don't know (✔)	0
Maximum HBVc knowledge score		4 of 4
Minimum HBVc knowledge score		0 of 4

***** HBV can be found in saliva but the concentration is very low compared to blood; direct injection through bites is required to transmit the virus via this medium.[1,2] Transmission has not been observed through sweat.[1]

References:

1. Canadian Center for Occupational Health and Safety (CCOHS). Hepatitis B. *OSH Facts Sheet* 2014. http://www.ccohs.ca/oshanswers/diseases/hepatitis_b.html (Accessed: 27th July 2015).
2. Centers for Disease Control and Prevention (CDC). When Someone Close to You has Viral Hepatitis. *Division of Viral Hepatitis* 2010. http://www.cdc.gov/hepatitis/HBV/PDFs/HepBWhenSomeoneClose.pdf (Accessed: 26th June 2015).

For peer review only

Appendix C: Frequency of Available Data and Missing Data from Completed Questionnaires, Federal Road Safety Corps, Kaduna Sector Command, Nigeria, June-July, 2015

Variable	Valid Sample Size	Available Data	Percentage	Missing Data	Percentage
Sex	**341**	**327**	**95.9**	**14**	**4.1**
Age	**341**	**338**	**99.1**	**3**	**0.9**
Duration of Service	**341**	**339**	**99.4**	**2**	**0.6**
Cadre	**341**	**341**	**100**	**0**	**0**
Ever heard of HBV infection?	**341**	**340**	**99.7**	**1**	**0.3**
Seriousness of HBV compared to HIV	**341**	**335**	**98.2**	**6**	**1.8**
Route of Transmission of HBV	**341**	**337**	**98.8**	**4**	**1.2**
Perception of Risk of exposure to HBV	**341**	**340**	**99.7**	**1**	**0.3**
Ever heard of hepatitis B vaccination?	**341**	**336**	**98.5**	**5**	**1.5**
Effectiveness of hepatitis B vaccination	**341**	**334**	**97.9**	**7**	**2.1**
Ever received hepatitis B vaccination?	**341**	**325**	**95.3**	**16**	**4.7**
Number of doses received	**198**	**198**	**100**	**0**	**0**
Recommended full dose of hepatitis B vaccine	**341**	**308**	**90.3**	**33**	**9.7**
Duration of protection from full-dose HBVc	**341**	**312**	**91.5**	**29**	**8.5**

STROBE 2007 (v4) Statement—Checklist of items that should be included in reports of *cross-sectional studies*

Section/Topic	Item #	Recommendation	Reported on page #
**Title and abstract**	1	(*a*) Indicate the study's design with a commonly used term in the title or the abstract	1
		(*b*) Provide in the abstract an informative and balanced summary of what was done and what was found	3,4
**Introduction**			
Background/rationale	2	Explain the scientific background and rationale for the investigation being reported	5,6
Objectives	3	State specific objectives, including any prespecified hypotheses	6
**Methods**			
Study design	4	Present key elements of study design early in the paper	6
Setting	5	Describe the setting, locations, and relevant dates, including periods of recruitment, exposure, follow-up, and data collection	6-8
Participants	6	(*a*) Give the eligibility criteria, and the sources and methods of selection of participants	8,9
Variables	7	Clearly define all outcomes, exposures, predictors, potential confounders, and effect modifiers. Give diagnostic criteria, if applicable	10,11
Data sources/ measurement	8*	For each variable of interest, give sources of data and details of methods of assessment (measurement). Describe comparability of assessment methods if there is more than one group	9-11
Bias	9	Describe any efforts to address potential sources of bias	8-10
Study size	10	Explain how the study size was arrived at	8
Quantitative variables	11	Explain how quantitative variables were handled in the analyses. If applicable, describe which groupings were chosen and why	10-12
Statistical methods	12	(*a*) Describe all statistical methods, including those used to control for confounding	10-12
		(*b*) Describe any methods used to examine subgroups and interactions	NA
		(*c*) Explain how missing data were addressed	12
		(*d*) If applicable, describe analytical methods taking account of sampling strategy	11,12
		(*e*) Describe any sensitivity analyses	NA
**Results**			

For peer review only

Participants	13*	(a) Report numbers of individuals at each stage of study—eg numbers potentially eligible, examined for eligibility, confirmed eligible, included in the study, completing follow-up, and analysed	12-17
		(b) Give reasons for non-participation at each stage	12
		(c) Consider use of a flow diagram	NA
Descriptive data	14*	(a) Give characteristics of study participants (eg demographic, clinical, social) and information on exposures and potential confounders	12,13
		(b) Indicate number of participants with missing data for each variable of interest	12
Outcome data	15*	Report numbers of outcome events or summary measures	15
Main results	16	(*a*) Give unadjusted estimates and, if applicable, confounder-adjusted estimates and their precision (eg, 95% confidence interval). Make clear which confounders were adjusted for and why they were included	15-17
		(*b*) Report category boundaries when continuous variables were categorized	10
		(*c*) If relevant, consider translating estimates of relative risk into absolute risk for a meaningful time period	NA
Other analyses	17	Report other analyses done—eg analyses of subgroups and interactions, and sensitivity analyses	NA
**Discussion**			
Key results	18	Summarise key results with reference to study objectives	17,18
Limitations	19	Discuss limitations of the study, taking into account sources of potential bias or imprecision. Discuss both direction and magnitude of any potential bias	21-23
Interpretation	20	Give a cautious overall interpretation of results considering objectives, limitations, multiplicity of analyses, results from similar studies, and other relevant evidence	18-21
Generalisability	21	Discuss the generalisability (external validity) of the study results	21,22
**Other information**			
Funding	22	Give the source of funding and the role of the funders for the present study and, if applicable, for the original study on which the present article is based	2

*Give information separately for cases and controls in case-control studies and, if applicable, for exposed and unexposed groups in cohort and cross-sectional studies.

Note: An Explanation and Elaboration article discusses each checklist item and gives methodological background and published examples of transparent reporting. The STROBE checklist is best used in conjunction with this article (freely available on the Web sites of PLoS Medicine at http://www.plosmedicine.org/, Annals of Internal Medicine at http://www.annals.org/, and Epidemiology at http://www.epidem.com/). Information on the STROBE Initiative is available at www.strobe-statement.org.

For peer review only

BMJ Open

Hepatitis B Vaccination Coverage, Knowledge and Socio-Demographic Determinants of Uptake in High-Risk Public Safety Workers in Kaduna State, Nigeria: A Cross-Sectional Survey

Journal:	*BMJ Open*
Manuscript ID	bmjopen-2017-015845.R1
Article Type:	Research
Date Submitted by the Author:	23-Feb-2017
Complete List of Authors:	Ochu, Chinwe; Ahmadu Bello University Teaching Hospital, Family Medicine<br>Beynon, Caryl; University of Liverpool
**Primary Subject Heading**:	Public health
Secondary Subject Heading:	Epidemiology, Occupational and environmental medicine, Infectious diseases
Keywords:	Infection control < INFECTIOUS DISEASES, Hepatitis B virus, Vaccination coverage, Public safety workers

SCHOLARONE™
Manuscripts

For peer review only

Hepatitis B Vaccination Coverage, Knowledge and Socio-Demographic Determinants of Uptake in High-Risk Public Safety Workers in Kaduna State, Nigeria: A Cross-Sectional Survey

Corresponding Author: Ochu, Chinwe Lucia

Department: Family Medicine

Institution: Ahmadu Bello University Teaching Hospital

City: Zaria

State: Kaduna State

Postal code: 810105

Country: Nigeria

Emails: lucia.ochu@online.liverpool.ac.uk; luciaochu@yahoo.com

Phone: +2348027413202

Co-Author: Beynon, Caryl M.

Department: Public Health

Institution: University of Liverpool (online)

Country: United Kingdom

Email: caryl.beynon@online.liverpool.ac.uk

Phone: NA

Word Counts: Abstract: 290; Main text: 4000 (excluding title page, statements, abstract, article summary, tables, acknowledgment and references).

Number of tables: 5

Number of references: 38

Number of supplementary files (appendices) for online only publication: 3

For peer review only

STATEMENTS

Contributorship: This study was carried out as a dissertation research by CLO under the close supervision of CMB, in partial fulfillment of the requirement for the award of the degree of Master of Public Health by the University of Liverpool, United Kingdom. CLO collected the data and conducted the analyses. These were reviewed by CMB. The manuscript was drafted by CLO and reviewed and revised by CMB. Both authors approved the final version for publication.

Competing Interests: None declared.

Funding: This research received no specific grant from any funding agency in the public, commercial or not-for-profit sectors.

Ethics Approval: This study was approved by the University of Liverpool's Ethics Committee and the Ethics Committee of Kaduna State Ministry of Health.

Participant Consent: Obtained

Data sharing: Extra data can be accessed via the Dryad data repository at http://datadryad.org/ with the doi:10.5061/dryad.545q0

For peer review only

For peer review only - http://bmjopen.bmj.com/site/about/guidelines.xhtml

ABSTRACT

Objectives: To estimate hepatitis B vaccination (HBVc) coverage, and knowledge and socio-demographic determinants of full-dose uptake in Federal Road Safety Corps (FRSC) members, Kaduna State, Nigeria in order to inform relevant targeted vaccination policies.

Design: A cross-sectional survey of FRSC members, Kaduna Sector Command.

Settings: Six randomly-selected Unit Commands under Kaduna Sector Command, Kaduna State, Nigeria.

Participants: Pilot-tested structured self-administered questionnaire was administered to 341 participants aged 18 years and above with ≥6 months of service between 17th June and 22nd July, 2015. Excluded were FRSC members in Road Safety (RS) 1 Zonal Command headquarters as the Zonal Command includes other States beyond the study scope.

Primary Outcome: HBVc status of participants categorized as 'not vaccinated' for uptake of <3 doses and 'vaccinated' for uptake of ≥3 doses.

Analysis: Descriptive analysis estimated HBVc coverage while logistic regression ascertained associations.

Results: Most participants were males, aged 30-39 years, with 3-10 years of service, and of Marshal cadre. HBVc coverage was 60.9% for ≥1 dose and 30.5% for ≥3 doses. Less than 47% of participants scored above the mean knowledge score for hepatitis B virus (HBV) and HBVc. Female sex (AOR 2.28, 95% CI 1.15-4.52, $p<0.05$), perceiving there to be an occupational risk of exposure to HBV (AOR 2.86, 95% CI 1.06-7.70, $p<0.001$), and increasing HBVc knowledge (AOR 2.68, 95% CI 1.83-3.92, $p<0.001$) were independent predictors of full-dose HBVc in FRSC members, Kaduna Sector Command.

For peer review only - http://bmjopen.bmj.com/site/about/guidelines.xhtml

Conclusions: HBVc coverage and knowledge are poor among FRSC members, Kaduna Sector Command. Educational intervention, geared towards improving FRSC members' knowledge of HBVc and perception of risk of occupational exposure to HBV, is recommended for these vulnerable public safety workers. Such enlightenment could be a cheap and easy way of improving HBVc coverage in the study population.

Keywords: infection control; hepatitis B virus; vaccination coverage; public safety workers

ARTICLE SUMMARY

Strengths and Limitations of this Study

- This is the first study to estimate hepatitis B vaccination coverage of public safety workers such as the Federal Road Safety Corps in Nigeria despite these workers being occupationally exposed to hepatitis B virus.
- The participating Unit Commands were randomly selected and the study had a high response rate hence minimizing selection bias and improving the generalizability of the research findings.
- Retrospective studies are prone to recall bias; this was mitigated in this study by omitting inconsistent data suggestive of guessing at the analysis stage.
- Researcher bias was mitigated by the use of a pre-validated data collection instrument and by pre-determining analytical strategies before data collection while confounding was minimized through multivariate analysis.
- Missing data made sample size in multivariate analysis less than the pre-research estimate, though the proportion analyzed constituted a good representation of the entire study population.

For peer review only - http://bmjopen.bmj.com/site/about/guidelines.xhtml

INTRODUCTION

Hepatitis B virus (HBV) is a highly infectious blood-borne pathogen usually transmitted via percutaneous or mucosal exposure to infected blood and body fluids.[1] HBV infection affects about one third of the world's population with >350 million persons being chronic carriers.[2,3] Sub-Saharan Africa and Southeast Asia have the highest prevalence of about 10-20% for chronic HBV.[2] HBV infection has heterogeneous outcomes: acute viral hepatitis, spontaneous clearance, or chronicity with its common fatal sequelae of hepatic cirrhosis and hepatocellular carcinoma (HCC).[3,4] Most adult-onset infections resolve spontaneously with only 5-10% resulting in chronic carriership.[2] Chronicity is commonly associated with early childhood exposures with an estimated 90% of perinatal transmissions becoming chronic infections.[4] Perinatal and horizontal transmissions are the predominant routes of HBV infection in hyperendemic settings.[5]

Hepatitis B vaccination (HBVc) is the most effective way of controlling HBV infection.[6] HBV control in sub-Saharan Africa targets mother-to-child transmissions via HBVc of children 0-5 years.[2,7] Though chronicity has been the major HBV outcome of public health interest, recent subtle transitions in the global mortality burden of HBV outcomes however demands readjustment of this focus. In a comparative systematic analysis of global disease burden, Lozano et al. demonstrated the trend in HBV-related outcomes between 1990 and 2010.[8] Though HBV-related HCC caused more deaths than acute HBV infection, the percentage increase in age-standardized death rates was about eleven times higher for acute HBV infection (29.2%) than for HBV-related HCC (2.6%) while death from HBV-related liver cirrhosis declined by 18.5%.[8] This growing mortality trend for acute HBV infection demands a renewed public health action in addressing this often neglected outcome of HBV. Prevention strategies for

For peer review only - http://bmjopen.bmj.com/site/about/guidelines.xhtml

HBV should also target those at high risk of acute infections. Public safety workers (PSWs) such as fire-fighters, correctional officers, rescue workers and emergency medical service providers with regular exposure to blood or body fluids, have similar risks as hospital-based healthcare workers (HCWs) of contracting HBV.[9] Besides, HCWs or PSWs, in the course of their duties, can infect children who consequently become chronic carriers. Controlling HBV infection in HCWs and PSWs is therefore of public health relevance. World Health Organization (WHO) prescribes universal HBVc of HCWs and PSWs with frequent blood-skin exposure.[10] A standard three-dose vaccine regimen, with the second and third doses given one month and six months apart from the initial dose respectively, is very effective in conferring immunity against HBV for ≥20 years.[1,11]

Nigeria is hyperendemic for HBV; Schweitzer et al. reported a pooled HBV prevalence estimate of 9.76% (95% confidence interval (CI) 9.59-9.93).[5] This hyperendemic status poses a great risk of occupational exposure to HBV for HCWs and PSWs with regular blood-skin contact, though this risk has not been estimated in any Nigerian study. The risk of transmission from infected blood is said to be 100 times more for HBV than for HIV in non-immune individuals.[11] HBVc became part of the Nigerian National Programme on Immunization for children 0-5 years in 2004.[12] Sub-optimal immunization coverage is however still a huge problem, especially in northern Nigeria.[12,13] To effectively control HBV in the Nigerian setting would therefore require plurality of approaches. Prevention of new infections in at-risk adults should complement prevention of perinatal transmissions. There is currently no universal HBVc programme for high-risk adults in Nigeria. Such adults, however, can access HBVc individually in primary healthcare centres at subsidized rates.

For peer review only - http://bmjopen.bmj.com/site/about/guidelines.xhtml

The Federal Road Safety Corps (FRSC) was established by the Federal Government of Nigeria in 1988 due to the high rate of road traffic crashes (RTCs) in the country.[14] Road safety functions of FRSC include rescue and emergency care of RTC victims and this brings them in regular contact with blood.[14] All FRSC members participate in rescue operations, though this is more frequent for the Marshal cadre. Crashed vehicles with broken glasses increase the risk of sharps injuries for these PSWs. This exposes them and the accident victims they rescue to a high risk of HBV infection in this hyperendemic setting. No study exists on HBVc coverage of PSWs in Nigeria. The objectives of this study were to estimate HBVc coverage, and knowledge and socio-demographic determinants of full-dose uptake in FRSC members, Kaduna State, Nigeria, in order to inform relevant targeted vaccination policies.

METHODS

Study Design: A quantitative cross-sectional survey of FRSC members, Kaduna Sector Command (KSC), Nigeria.

Setting and Target Population: Kaduna State is the third most populous State in Nigeria and has 3 senatorial zones with 23 local government areas (LGAs).

The FRSC is divided into 12 Zonal Commands; each Zonal Command has Sector Commands which are sub-divided into Unit Commands (UCs).[15] There are currently 204 UCs in Nigeria.[15] The first 11 UCs are located in the KSC with the KSC headquarters making them 12; these cover the entire 23 LGAs in Kaduna State (Table 1).

For peer review only - http://bmjopen.bmj.com/site/about/guidelines.xhtml

Table 1: Location, Coverage and Staff Distribution of Unit Commands (UCs) of FRSC, Kaduna Sector Command, Nigeria, June-July, 2015.

Commands	Designation	Staff Strength			Number of LGAs Covered	Location (LGA)
		Cadre				
		Officer	Marshal	Total		
Kaduna Sector Command (KSC) Headquarters	*RS1.1	46	118	164	2	Kaduna North
Kafanchan UC	RS1.11	15	44	59	4	Jama’a
Birnin Gwari UC	RS1.12	17	35	52	1	Birnin Gwari
Zaria UC	RS1.13	24	66	90	5	Sabon Gari
Saminaka UC	RS1.14	10	36	46	1	Lere
Sabon Tasha UC	RS1.15	16	52	68	1	Chikun
Kakau UC	RS1.16	18	66	84	2	Chikun
Birnin Yero UC	RS1.17	15	44	59	1	Igabi
Gwantu UC	RS1.18	8	31	39	2	Sanga
Katari UC	RS1.19	19	37	56	1	Kachia
Kachia UC	RS1.110	10	26	36	2	Kachia
Tashan Yari UC	RS1.111	10	26	36	1	Makarfi
	**Total**	**208**	**580**	**789**	**23**	

***RS: Road Safety**

KSC is one of the four Sector Commands that make up the RS1 Zonal Command whose headquarters is in Kaduna. Two major cadres exist in FRSC: Officer and Marshal, though the latter is sub-divided into Marshal Inspectorate and Road Marshal Assistant. At the time of this study, there were 789 FRSC members in KSC, 26% of which were officers and 74% Marshals. The study was conducted in six randomly selected UCs: KSC headquarters, Saminaka, Kakau, Gwantu, Katari, and Kachia.

For peer review only

For peer review only - http://bmjopen.bmj.com/site/about/guidelines.xhtml

Inclusion and Exclusion Criteria: Only FRSC members in KSC aged ≥18 years with ≥6 months of service were included in the study. This ensured that only adults long enough in service to be made aware of the risk of HBV were surveyed. FRSC members in RS1 Zonal Command headquarters were excluded from the study as the Zonal Command includes other States beyond the study scope.

Sample Size: This was estimated using the formula for cross-sectional surveys: $n = 1.96^2 \times p(1-p)/d^2$, where n is the required sample size, p is prevalence estimate of HBVc in previous studies, and d is precision or acceptable error margin (5%).[16] Ogoina et al.'s prevalence estimate of 36.2% in a survey of 290 HCWs in Nigeria[17] was used as proxy since there is no existing study on PSWs in Nigeria. Anticipating a lower prevalence rate among non-HCWs with expectedly lower level of awareness of HBVc, 30% prevalence was assumed. ($N = 1.96^2 \times 0.3(1-0.3)/0.5^2 = 323$). Using 24% as anticipated non-response rate (q),[17] a final sample size of 425 was estimated using the formula: $N_f = N_s/1\text{-}q$, where N_f is the final sample size and N_s the initial sample size.[18]

Sampling: The sampling frame was a list of the 12 UCs from the KSC headquarters. Each UC was considered a cluster. Clusters were randomly selected using a computer-generated set of random numbers until sample size was achieved. This simple random selection of clusters was to ensure representativeness of selected UCs.[19] Six UCs were selected for the study. All FRSC members in the selected UCs were targeted for questionnaire distribution.

Data Collection: UCs of FRSC have compulsory weekly meetings. Permission was obtained for data collection at these meetings. The UCs were informed of the research prior to visits. Data were collected between 17th June and 22nd July, 2015. Participant information sheet (PIS) was

For peer review only - http://bmjopen.bmj.com/site/about/guidelines.xhtml

reviewed with the staff with emphasis on voluntary participation, anonymity and confidentiality of collected data. Inclusion criteria and implied consent were further explained; completion of questionnaire was considered consent to participate. Participants were asked to seal completed questionnaires in the given envelopes and drop them in a common collection box provided by the researcher. This was to ensure anonymity. Those unwilling to participate were asked to drop the sealed uncompleted questionnaires in the box alongside participants. Non-respondents were therefore not identified during data collection. Two UCs (KSC Headquarters & Kakau) were re-visited in subsequent meetings due to poor initial attendance. Routine attendance lists taken by the UCs at the initial meetings were used to prevent re-participation of previous participants.

Instruments: Due to paucity of studies on the research topic, accessing a pre-validated questionnaire for the study was difficult. After an extensive literature search, only Al-Hussami's "Hepatitis B Vaccine Knowledge and Acceptance" questionnaire could be found.[20] This questionnaire has been used for HCWs in United States. It was validated in two pilot studies with testing for inter-reliability but the test statistic was not reported.[20] There were 44 multiple choice questions including some open-ended ones. A structured anonymous self-administered questionnaire was adapted from this questionnaire for the present study (appendix A). Only questions relevant to the research questions were selected. Questions were simplified to suit the literacy status of the study population. The adapted study questionnaire contained 17 questions that elicited information on demographics (sex, age, duration of service, cadre and rank), HBV knowledge and perception of risk of exposure, and HBVc knowledge and status. Though rank was obtained, this was not included in analysis since it mirrors cadre. The questionnaire was pilot-tested on FRSC members in RS1 Zonal Command headquarters.

Statistical Analysis: Table 2 describes the variables in the study.

For peer review only - http://bmjopen.bmj.com/site/about/guidelines.xhtml

Table 2: Description of Variables in the Study, FRSC, KSC, Nigeria, June-July, 2015.

Variable	Description	Type of Data
Independent Variables		
Sex	This was the gender of study participants categorized as either male or female	Nominal
Age	This variable ascertained the age of participants on their last birthday. It was categorized to enhance anonymity from 18 years which is the age definition of commencement of adulthood to ≥50 years which marks the age before retirement from Nigerian Civil Service at 60 years. The categories included: 18-29 years; 30-39 years; 40-49 years; ≥50 years	Ordinal
Duration of Service	This variable elicited how long a respondent had been in service with the Federal Road Safety Corps. It was categorized into: 6 months-2 years (probation period in civil service); 3- 10 years; 11 years to 19 years; and ≥20 years (close to retirement by service year at 35 years).	Ordinal
Cadre	This ascertained the official class of participant based on position and seniority in office. There were two major categories: Officers and Marshals with the latter sub-categorized into Marshal Inspectorate and Field Marshal Assistant in a descending order. It also signified educational qualification order with the least educated being the Field Marshal Assistant.	Nominal/Ordinal
Risk Perception	This ascertained the level of perception of occupational risk of exposure to HBV by respondents. It was initially categorized into: No risk of exposure, low risk of exposure, moderate risk of exposure, high risk of exposure, and I don't know. This was later dichotomized for further analysis by merging the 'I don't know' group with the 'no risk' group to form a 'no risk perceived' category	Nominal/Ordinal

For peer review only

For peer review only - http://bmjopen.bmj.com/site/about/guidelines.xhtml

Table 2: Description of Variables in the Study, FRSC, KSC, Nigeria, June-July, 2015.

Variable	Description	Type of Data
	with the rest forming the 'risk perceived' category.	
Hepatitis B Virus (HBV) Knowledge Score	This variable sought to estimate the level of knowledge of basic information on HBV. It includes questions on HBV awareness, seriousness compared to HIV, and route of transmission. For each participant, the number of questions answered correctly was noted as the score (see scoring table in appendix B).	Continuous
Hepatitis B Vaccination (HBVc) Knowledge Score	This measured the level of basic knowledge of HBVc among participants. It comprised questions on HBVc awareness, effectiveness, recommended full dosage and duration of protection from full-dose vaccination. For each participant, the number of questions answered correctly was noted as the score (appendix B).	Continuous
Dependent Variable		
HBVc Status	Information was elicited on whether participant had ever received HBVc and the number of doses received. Descriptive analysis was done using these data. Dichotomization of data was also done for logistic regression analysis. Since only those with ≥3 doses of HBVc uptake are considered fully protected,[11] those with ≥3 doses were labeled 'vaccinated' and the rest 'not vaccinated'. This was noted as the HBVc status of each participant.	Nominal

All analyses were conducted using SPSS version 21. Descriptive analysis ascertained frequencies and distributions of data. Histograms showed both HBV knowledge and HBVc knowledge scores to be normally distributed, hence their mean and standard deviations (SD) were calculated

For peer review only - http://bmjopen.bmj.com/site/about/guidelines.xhtml

as was the percentage of participants scoring above the mean scores. Since the outcome variable (HBVc status) was binary, logistic regression analysis was used in testing for associations with the independent variables (table 2).[21] To mitigate confounding, univariate analyses were first carried out and the variables identified as significantly associated ($p<0.05$) with HBVc status were included in the multivariate analysis for independent predictors of full-dose HBVc.[21] Adjusted odds ratios (AOR) with 95% CI for each variable was computed and significance level set at $p<0.05$. Missing data on each variable were excluded in the analysis of the variable.

RESULTS

There were 354 questionnaires distributed in the six UCs sampled from FRSC, KSC. Six questionnaires were discarded for having missing data on up to 3 of the independent variables or on the dependent variable and ≥2 independent variables. Seven questionnaires were submitted blank. In all, 341 completed questionnaires were included for analysis, giving a response rate of 96.3%. Appendix C shows percentage of missing data for each of the 14 questions analyzed. Missing data were most frequent on the question on recommended dose of vaccine (9.7%; 33/341) followed by that on the duration of protection from full-dose HBVc (8.5%; 29/341). All participants provided data on cadre. Most respondents were males; aged 30-39 years; had worked between 3-10 years with FRSC; and were of Marshal Cadre (table 3).

For peer review only - http://bmjopen.bmj.com/site/about/guidelines.xhtml

Table 3: Socio-Demographic Characteristics of Study Sample of FRSC Members, KSC, Nigeria, June-July, 2015.

**Variable**	**Frequency**	**Percentage**
Sex (n=327)		
Male	260	79.5
Female	67	20.5
Age (n=338)		
18-29 years	64	18.9
30-39 years	167	49.4
40-49 years	87	25.7
≥50 years	20	5.9
Duration of Service (n=339)		
6 months-2 years	36	10.6
3-10 years	188	55.5
11-19 years	87	25.7
≥20 years	28	8.3
Cadre (n=341)		
Officer	96	28.2
Marshal	245	71.8
-Marshal Inspectorate	111	32.6
- Field Marshal Assistant	134	39.3

HBV Knowledge: The mean total number of correct answers to HBV knowledge questions was 3.0 out of 6.0 (SD 1.5). Only 46% (157/341) of participants scored above the mean. The proportion of correct answers to HBV knowledge questions ranged from 2.1% (7/337) on route of transmission of HBV to 93.2% (317/340) on having ever heard of HBV. Approximately 22.6% (76/337) of respondents answered 'I don't know' to the question pertaining to the route of transmission of HBV and this response was the most frequent. Merely 2.1% (7/337) correctly identified contact with infected blood and blood-contaminated body fluid as routes of transmission of HBV. Only 4 participants (1.2%; n=341) answered all 6 HBV knowledge

For peer review only - http://bmjopen.bmj.com/site/about/guidelines.xhtml

questions correctly while 16 (4.7%, n=341) answered none correctly. HBV infection was perceived as more serious than HIV by most respondents (56.7%; 190/335) while about 3.0% (10/335) felt it was less serious than HIV. While 20.6% (69/335) claimed no knowledge of the seriousness of HBV compared to HIV, 19.7% (66/335) ascribed equal severity to the two.

HBVc Knowledge: The mean number of correct answers to HBVc questions was 2.0 out of 4.0 (SD 1.1). Approximately 42.2% (144/341) of participants had scores higher than the mean score. All four questions on HBVc were answered correctly by only 4.1% (14/341) of participants while no correct answer was given by 11.7% (40/341). Rate of correctness ranged from 6.1% (19/312) on question on duration of protection from full-dose HBVc to 86.6% (291/336) on having ever heard of HBVc. Most respondents (62.9%; 210/334) described HBVc as very effective. While 6.9% (23/334) rated it slightly effective, 2.7% (9/334) felt it was not effective at all and 27.5% (92/334) indicated not knowing its effectiveness. Roughly 54.9% (169/308) of respondents correctly identified recommended full HBVc dose as ≥3 doses while 1.6% (5/308) and 3.9% (12/308) thought it was 1 dose and 2 doses respectively. Up to 39.6% (122/308) indicated not knowing the recommended full dose of HBVc.

Perception of Risk of Occupational Exposure to HBV: While most respondents (55.3%; 188/340) rated themselves at high risk of occupational exposure to HBV, 22.4% (76/340) did not know their risk status. Whereas 5.3% (18/340) of respondents considered themselves at no risk of exposure to HBV, 5.9% (20/340) and 11.2% (38/340) rated themselves at low and moderate risks of exposure respectively. After dichotomizing this variable into 'no risk perceived' and 'risk perceived' categories, 72.4% (246/340) had some level of risk perception while 27.6% (94/340) had no risk perception for HBV.

For peer review only - http://bmjopen.bmj.com/site/about/guidelines.xhtml

For peer review only

HBVc Coverage: Of the 341 participants, 6 did not provide data on their HBVc status. Ten others answered ‘yes’ to having ever received HBVc but omitted the number of doses received and were therefore inputted as missing data. Only 325 respondents (95.3%) were included in the sub-analysis. Roughly 60.9% (198/325) of the respondents affirmed having ever received ≥1 dose of HBVc and 50.0% of these (99/198) had received ≥3 doses resulting in full-dose coverage of 30.5% (99/325) among the respondents. Approximately 39.1% (127/325) of respondents had never received HBVc. Together with the 99 participants with <3 doses, 69.5% (226/325) were classified ‘not vaccinated’ while 30.5% (99/325) were labeled ‘vaccinated’.

Logistic Regression Analyses: All the variables were significantly associated with HBVc on univariate analyses (table 4) and were included in the multivariate analysis for independent predictors of full-dose HBVc uptake (table 5). Being female was associated with about twice the likelihood of having received full-dose HBVc (table 5). When risk perception was analyzed as a dichotomous variable (‘no risk perceived’ versus ‘risk perceived’), those with any level of risk perception for occupational exposure to HBV were about 3 times more likely to have received full-dose HBVc than those without risk perception for HBV (table 5). Though the odds of being fully vaccinated increased with duration of service, this was not statistically significant. While HBV knowledge was not a significant predictor of full-dose HBVc, knowledge of HBVc was significantly associated with full-dose HBVc with each unit increase in number of correct answers being associated with up to three times increased likelihood of being fully vaccinated (table 5).

For peer review only - http://bmjopen.bmj.com/site/about/guidelines.xhtml

Table 4: Univariate Binary Logistic Regression Analyses Showing Unadjusted Odds Ratios of the Association between Independent Variables and Full-Dose Hepatitis B Vaccination Uptake among FRSC Members, KSC, Nigeria, June-July, 2015

Independent Variable	Sample Size	Odds Ratio	95% CI (p-value)
Sex	313		
Male		1	
Female		2.66	1.51-4.70 (0.001)
Age	323		
18-29 years		1	
30-39 years		2.08	0.97-4.44 (0.059)
40-49 years		3.30	1.47-7.40 (0.004)
≥50 years		5.67	1.84-17.50 (0.003)
Duration of service	323		
6 months-2 years		1	
3-10 years		5.69	1.31-24.72 (0.020)
11-19 years		9.48	2.12-42.35 (0.003)
≥20 years		13.39	2.61-68.56 (0.002)
Cadre	325		
Officer		1	
Marshal Inspectorate		1.69	0.92-3.09 (0.091)
Road Marshal Assistant		2.10	1.18-3.74 (0.012)
Risk Perception for Occupational Exposure to HBV	324		
I don't know		1	
No risk		3.92	0.78-19.63 (0.096)
Low risk		11.33	2.94-43.63 (<0.001)
Moderate risk		9.61	2.85-32.43 (<0.001)
High risk		11.07	3.87-31.70 (<0.001)
Risk perception for Occupational Exposure to HBV	324		
No risk perceived		1	
Risk perceived		7.39	3.27-16.71 (<0.001)
HBV Knowledge Score	325	1.37	1.15-1.62 (<0.001)
HBVc Knowledge Score	325	2.97	2.16-4.08 (<0.001)

For peer review only

For peer review only - http://bmjopen.bmj.com/site/about/guidelines.xhtml

Table 5: Multivariate Binary Logistic Regression Analysis for Independent Predictors of Full-Dose Hepatitis B Vaccination Uptake among Members of FRSC, KSC, Nigeria, June-July, 2015.

Independent Variable	Adjusted Odds Ratio n=309	95% CI (p-value)
Sex		
Male	1	
Female	2.28	1.15-4.52 (0.019)
Age		
18-29 years	1	
30-39 years	1.40	0.47-4.18 (0.542)
40-49 years	0.99	0.28-3.55 (0.987)
≥50 years	1.08	0.20-5.76 (0.931)
Duration of service		
6 months-2 years	1	
3-10 years	2.12	0.39-11.41 (0.384)
11-19 years	2.73	0.45-16.59 (0.276)
≥20 years	5.25	0.68-40.47 (0.112)
Cadre		
Officer	1	
Marshal Inspectorate	1.60	0.77-3.33 (0.208)
Road Marshal Assistant	0.87	0.41-1.85 (0.720)
Risk Perception for Occupational Exposure to HBV		
I don't know	1	
No risk	2.93	0.47-18.41 (0.251)
Low risk	7.12	1.47-34.47 (0.015)
Moderate risk	4.50	1.03-19.63 (0.045)
High risk	3.90	1.08-14.09 (0.038)
Risk perception for Occupational Exposure to HBV		
No risk perceived	1	
Risk perceived	2.86	1.06-7.70 (<0.001)
HBV Knowledge Score	1.03	0.80-1.31 (0.843)
HBVc Knowledge Score	2.68	1.83-3.92 (<0.001)

For peer review only

In summary, full-dose HBVc was 30.5% while ≥1 dose coverage was 60.9%. Female sex, perceiving their occupation as conveying a risk of HBV, and increasing HBVc knowledge were significant independent predictors of full-dose HBVc uptake among members of FRSC in Kaduna State, Nigeria.

DISCUSSION

Like all other studies on HCWs in Nigeria,[22-25] this study has further demonstrated high HBVc initiation rate (60.9%) with low completion rate (30.5%). This completion rate implies that only 30.5% of members of the FRSC, KSC were adequately protected against HBV infection.[11] This means that almost 70% of these rescue workers perform their duties without adequate protection from HBV. This also places the accident victims whom they seek to rescue (children inclusive) at risk of infection with HBV from infected FRSC members. This could lead to an unbroken cycle of infectivity, morbidity and mortality from HBV in a nation still struggling with the HBV scourge. Poor uptake of HBVc among those at occupational risk of exposure to HBV in Nigeria is a common observation across studies.[22-28] Adoption of a universal HBVc policy in the country for HCWs and vulnerable PSWs could improve vaccine uptake. FRSC members are exposed to blood and sharps injuries from accident scenes. In a nation with high prevalence of chronic HBV,[5] HBVc coverage of 30.5% for FRSC members, KSC is low. A sero-prevalence study to investigate the actual prevalence of HBV in these PSWs for appropriate intervention is therefore recommended.

Among FRSC members, females are 2.28 times more likely to be fully vaccinated against HBV compared to their male counterparts. Osazuwa-Peters et al. observed a similar but insignificant female preponderance in HBVc among dental professionals in Edo State, Nigeria.[29]

For peer review only - http://bmjopen.bmj.com/site/about/guidelines.xhtml

Contrarily, Adekanle et al., in their survey of HCWs in Ile-Ife, Nigeria, observed 1.8 times increased chances of males receiving HBVc compared to females,[27] although this finding may be due to males in the study being mainly doctors who had the advantage of professional knowledge of HBVc. In this present study, females could probably be exposed to HBVc knowledge during antenatal hospital visits and while taking their children for immunization.

More than 50% of respondents perceived themselves at high risk of occupational exposure to HBV. Disturbingly though, close to a quarter of participants claimed ignorance of their risk status. Together with those who perceived themselves to be at no risk, 27.6% of FRSC members had no risk perception for occupational exposure to HBV while 72.4% perceived themselves at risk. This falls within the range of 30% to 78% risk perception observed in studies among HCWs in Nigeria.[22,28] Despite high risk perception rate, having approximately 28% with no risk perception for HBV is quite disconcerting from a public health perspective considering the blood-skin exposure that rescue of accident victims could entail. Those who perceived themselves at risk of occupational exposure to HBV were three times more likely to be vaccinated than those without risk perception. All risk categories had higher odds of vaccination compared to those without risk perception. It is therefore important that FRSC members understand the risk of exposure to HBV (even if they feel it is low) as this appears to increase their likelihood of getting vaccinated.

Knowledge of HBV and HBVc among study participants was poor. Less than 47% of participants scored above the mean knowledge scores for HBV and HBVc. Knowledge was poorest for the route of transmission of HBV and duration of protection from full-dose HBVc. Not knowing the route of transmission of HBV means that FRSC members might take inadequate precautions against HBV during rescue operations. It could also lead to

For peer review only

stigmatization of FRSC members already infected with HBV due to wrong assumption of infectivity through casual contact with sweat and saliva. HBV knowledge score was however not an independent predictor of HBVc in the study. This conflicts with Adekanle et al.'s observation of twice increased likelihood of complete HBVc among those with good knowledge of HBV in their survey of HCWs in Ile Ife, Nigeria.[27] Their study though did not elicit information on HBVc knowledge, a potential confounder in the demonstrated association.

Despite 62.9% of respondents describing HBVc as very effective, only 6.1% knew that full-dose HBVc gives protection for ≥20 years. Knowing that receiving ≥3 doses of the vaccine can provide lifetime protection from HBV could incentivize full-dose uptake. HBVc knowledge was the most significant and precise independent predictor of full-dose HBVc in this study. This contradicts Ogoina et al.'s finding of no significant association between HBVc knowledge and full-dose vaccination among HCWs in two tertiary hospitals in Nigeria.[17] However, they did not ascertain knowledge of vaccine effectiveness and duration of protection from full-dose vaccination.

Age, cadre and duration of service were not significantly associated with HBVc in this study. Izegbu et al. observed more likelihood of HBVc with decreasing age,[30] while Sofola et al. associated increasing cadre with HBVc.[31] In another instance, longer duration of service was demonstrated to be associated with HBVc.[32] All these studies were among health professionals who expectedly have professional exposure to HBV and HBVc knowledge unlike the present study population.

Educational programme towards improvement of HBV and HBVc knowledge, and risk perception among FRSC members is a recognized relevant public health intervention from this

For peer review only - http://bmjopen.bmj.com/site/about/guidelines.xhtml

study. The programme can be included in the schedules of the already existing compulsory weekly in-house training/manpower development of staff and in the routine basic training programme for new staff. Such enlightenment would be a cheap and easy intervention to improve HBVc uptake. Existing evidence on the positive impact of educational intervention on vaccine uptake is however weak.[33] The educational intervention should therefore be rigorously evaluated to ascertain its impact on HBVc uptake in FRSC members.

Study Strengths and Limitations:

This was a descriptive cross-sectional survey which limits its suitability for demonstrating temporal relationships between explanatory and outcome variables.[34] It nevertheless shows independent associations useful in understanding predictors of full-dose HBVc in this study population so as to inform relevant public health interventions. Recall bias is another limitation of this retrospective study design as participants may not have remembered accurately their vaccination history thereby introducing information bias.[35] This was mitigated at the analysis stage by omitting inconsistent data suggestive of guessing. Simple random sampling using the staff register would have yielded a more representative sample;[21] disproportionate distribution of such socio-demographic variables as sex and cadre in the study population made this unfeasible. The response rate of 96.3% was however impressive and minimizes selection bias thereby enhancing the generalizability of research findings by improving the external and internal validity of the study.[36] The presence of Unit Commanders and other senior members of staff at the meetings during data collection and their participation in the research could have contributed to the high response rate. Subordinates who ordinarily may have declined participation could have felt a psychological obligation to participate with their bosses. This power influence was minimized by the use of PIS which emphasized voluntary participation, and

For peer review only - http://bmjopen.bmj.com/site/about/guidelines.xhtml

by anonymous data collection procedures. While anonymity and self-administration of questionnaire could lessen social desirability bias, the use of social desirability scale would have been more appropriate in demonstrating this bias for appropriate statistical control.[37] Researcher bias was minimized by the use of a pre-validated questionnaire and by pre-determining analytical strategies before data collection.[21] Possible exchange of information among participants could have introduced information bias. This was mitigated by the presence of the researcher during data collection with prior emphasis on non-communication between participants. Some participants who claim ignorance of HBV might have a different designation for the disease in the local language. This could bias the findings on HBV knowledge. Though the study's questionnaire was adapted and pilot-tested to reflect the study context, it was not tested for inter-rater reliability and validity within the study population. Missing data reduced the sample size for the multivariate analysis from 341 to 309. This was less than the pre-study estimate (323) and could lack sufficient power to detect significant associations, hence predisposing to type II error.[21] It however constitutes a randomized 39% (309/789) of the study population, which is a good representation.[38] Confounding, a known menace in observational studies, was minimized at the analytical stage through multivariate logistic regression.[21] The research estimates on the association of varied levels of risk perception with full-dose HBVc had very wide 95% CIs. This could be due to random errors in the sample.[36] A larger sample size in future studies could yield more precise estimates.

CONCLUSION

Controlling HBV transmission is an important public health issue internationally and in Nigeria where the virus is hyperendemic. HBV infection is a preventable disease and prevention is best achieved with HBVc.[6] FRSC members come in regular contact with blood and are at risk of

For peer review only - http://bmjopen.bmj.com/site/about/guidelines.xhtml

contracting HBV. HBVc coverage among FRSC members in Kaduna State, Nigeria is low (30.5%). Knowledge of HBV and HBVc is poor in this study population. Female sex, perceiving there to be an occupational risk of exposure to HBV, and increasing HBVc knowledge are independent predictors of HBVc uptake among FRSC members, KSC. Educational intervention aimed at improving awareness of the occupational risk of HBV and the importance of HBVc, is required to improve HBVc coverage among this vulnerable group of PSWs. Recommended future studies include: a qualitative study to ascertain FRSC members' perception of HBVc and subjective reasons for non-uptake of the vaccine; a sero-prevalence study to determine the actual immune status of FRSC members in KSC and estimate the prevalence of HBV in this study group for appropriate intervention; and validation of questionnaire in the Nigerian context with pretesting and retesting for reliability.

ACKNOWLEDGMENTS: University of Liverpool is acknowledged for providing the platform for this research. We thank the Commonwealth Scholarship Commission for paying the tuition fee for the MPH study of the corresponding author thereby enabling this dissertation research. We appreciate the staff and management of the Federal Road Safety Corps, Kaduna Sector Command for their cooperation and participation in this study.

REFERENCES

1. World Health Organization (WHO). Hepatitis B. *WHO* 2015. http://www.who.int/mediacentre/factsheets/fs204/en/ (Accessed: 23rd July 2015).
2. Meireles LC, Marinho RT, Van Damme P. Three Decades of Hepatitis B Control with Vaccination. *World J Hepatol*. 2015;7:2127-32. https://www.ncbi.nlm.nih.gov/pmc/articles/PMC4550866/ (Accessed: 7 February 2017).

For peer review only - http://bmjopen.bmj.com/site/about/guidelines.xhtml

3. Lemoine M, Eholie S, Lacombe K. Reducing the Neglected Burden of Viral Hepatitis in Africa: Strategies for a Global Approach. *Journal of Hepatology* 2015;62:469-76.

4. Mohamadkhani A, Katoonizadeh A, Poustchi H. Immune-Regulatory Events in the Clearance of HBsAg in Chronic Hepatitis B: Focuses on HLA-DP. *Middle East J Dig Dis* 2015;7:5-13. https://www.ncbi.nlm.nih.gov/pmc/articles/PMC4293802/ (Accessed: 8 February 2017).

5. Schweitzer A, Horn J, Mikolajczyk RT, et al. Estimations of Worldwide Prevalence of Chronic Hepatitis B Virus Infection: A Systematic Review of Data Published between 1965 and 2013. *The Lancet* 2015;386:1546-55.

6. Lavanchy D. Chronic Viral Hepatitis as a Public Health Issue in the World. *Best Practice & Research Clinical Gastroenterology* 2008;22(6):991-1008.

7. Stockdale AJ, Geretti AM. Chronic Hepatitis B Infection in Sub-Saharan Africa: A Grave Challenge and a Great Hope. *Trans R Soc Trop Med Hyg* 2015;109:421-22.

8. Lozano R, Naghavi M, Foerman K, et al. Global and Regional Mortality from 235 Causes of Death for 20 Age Groups in 1990 and 2010: A Systematic Analysis for the Global Burden of Disease Study 2010. *Lancet* 2012;380:2095-128.

9. Rischitelli G, Harris J, McCauley L, et al. The Risk of Acquiring Hepatitis B or C among Public Safety Workers- A Systematic Review. *Am J Prev Med* 2001;20:299-306.

10. World Health Organization (WHO). Table 1: Summary of WHO Position Papers- Recommendations for Routine Immunization. *Immunization, Vaccines and Biologicals* 2014. http://www.who.int/immunization/policy/immunization_tables/en/ (Accessed: 20 February 2015).

For peer review only

11. Centers for Disease Control and Prevention (CDC). Immunization of Health-Care Personnel: Recommendations of the Advisory Committee on Immunization Practices (ACIP). *Morbidity and Mortality Weekly Report (MMWR): Recommendations and Reports* 2011;60(RR07):1-45. http://www.cdc.gov/mmwr/preview/mmwrhtml/rr6007a1.htm (Accessed: 12th July 2015).

12. Ophori EA, Tula MY, Azih AV et al. Current Trends of Immunization in Nigeria: Prospect and Challenges. *Trop Med health* 2014;42:67-75.

13. Gunnala R, Ogbuanu IU, Adegoke OJ, et al. Routine Vaccination Coverage in Northern Nigeria: Results from 40 District-Level Cluster Surveys, 2014-2015. *PLoS One* 2016;11:e0167835. doi: 10.1371/journal.pone.0167835.

14. Chidoka O. The Lead Agency Concept: Federal Road Safety Corps, Nigeria Experience. Paper presented at the African Road Safety Management Workshop; 2014; Freetown, Sierra Leone. http://www.ssatp.org/sites/ssatp/files/pdfs/Events/2014/Presentation%20Nigeria.pdf (Accessed: 19 April 2015).

15. Federal Road Safety Corps (FRSC). Official Website. *Federal Government of Nigeria* 2015. http://frsc.gov.ng/ (Accessed: 22 February 2015).

16. Charan J, Biswas, T. How to Calculate Sample Size for Different Study Designs in Medical Research? *Indian J Psychol Med* 2013;35:121-6.

17. Ogoina D, Pondei K, Adetunji B, et al. Prevalence of Hepatitis B Vaccination among Health Care Workers in Nigeria in 2011-12. *Int J Occup Environ Med* 2014;5:51-56.

For peer review only

For peer review only - http://bmjopen.bmj.com/site/about/guidelines.xhtml

18. Whitley E, Ball J. Statistics Review 4: Sample Size Calculations. *Crit Care* 2002;6:335-41.

19. Hammer GP, Prel J-B, Blettner M. Avoiding Bias in Observational Studies. *Dtsch Arztebl Int* 2009;106:664-8.

20. Al-Hussami M. Knowledge and Acceptance of Hepatitis B Vaccine. *The Internet Journal of Healthcare Administration* 2001;2. https://ispub.com/IJHCA/2/1/5725 (Accessed: 20th April 2015).

21. Bruce N, Pope D, Stanistreet D. Quantitative Methods for Health Research: A practical Interactive Guide to Epidemiology and Statistics. Chichester, UK: Wiley 2008.

22. Ibekwe RC, Ibeziako N. Hepatitis B Vaccination Status among Health Workers in Enugu. *Niger J Clin Pract* 2006;9:7-10.

23. Fatusi AO, Fatusi OA, Esimai AO, et al. Acceptance of Hepatitis B Vaccine by Workers in a Nigerian Teaching Hospital. *East Afr Med J* 2000;77:608-12.

24. Okwara EC, Enwere OO, Diwe CK, et al. Theatre and Laboratory Workers' Awareness of and Safety Practices against Hepatitis B and C Infection in a Suburban University Teaching Hospital in Nigeria. *Pan Afr Med J* 2012;13:2-6.

25. Paul N, Peterside O. Hepatitis B Vaccination Rate among Medical Students at the University of Port Harcourt Teaching Hospital (UPTH). *World Journal of Vaccines* 2015;55:1-7. http://dx.doi.org/10.4236/wjv.2015.51001 (Accessed: 22 June 2015).

26. Azodo CC, Ehizele AO, Uche I, Erhabor P. Hepatitis-B Vaccination Status among Dental Surgeons in Benin City, Nigeria. *Ann Med Health Sci Res* 2012;2:24-8.

27. Adekanle O, Ndububa DA, Olowookere SA, et al. Knowledge of Hepatitis B Virus Infection, Immunization with Hepatitis B Vaccine, Risk Perception, and Challenges to

For peer review only - http://bmjopen.bmj.com/site/about/guidelines.xhtml

Control Hepatitis among Hospital Workers in a Nigerian Tertiary Hospital. *Hepatitis Research and Treatment* 2015;2015:1-6 [Online]. Available from: http://dx.doi.org/10.1155/2015/439867 (Accessed: 23 May 2015).

28. Adebamowo CA, Odukogbe AA, Ajuwon AJ. Knowledge, Attitude, and Practices Related to Hepatitis B Virus Infection among Nigerian Obstetricians and Midwives. *J Obstet Gynaecol* 1998;18:528-32.
29. Osazuwa-Peters N, Azodo CC, Ehizele AO, Obuekwe ON. Gender Differences in Characteristics, Occupational Exposure, and Infection Control Practices among Dental Professionals in Edo State, Nigeria. *South Afr J of Epidemiol Infect* 2012;27:61-5.
30. Izegbu MC, Amole OO, Ajayi GO. Attitudes, Perception and Practice of Workers in Laboratories in the Two Colleges of Medicine and their Teaching Hospitals in Lagos State, Nigeria as Regards Universal Precaution Measures. *Biomedical Research* 2006;17:49-54.
31. Sofola OO, Uti OG. Hepatitis B Virus Infection and Prevention in the Dental Clinic: Knowledge and Factors Determining Vaccine Uptake in a Nigerian Dental Teaching Hospital. *Nig Q J Hosp Med* 2008;18:145-48.
32. Olubuyide IO, Ola SO, Aliyu B, et al. Hepatitis B and C in Doctors and Dentists in Nigeria. *Q J Med* 1997;90:417-22.
33. Dube E, Gagnon D, MacDonald NE, the SAGE Working Group on Vaccine Hesitancy. Strategies Intended to Address Vaccine Hesitancy: Review of Published Reviews. *Vaccine* 2015;33:4191-203.
34. Carlson MDA, Morrison RS. Study Design, Precision, and Validity in Observational Studies. *J Palliat Med* 2009;12:77-82.

For peer review only - http://bmjopen.bmj.com/site/about/guidelines.xhtml

35. Neugebauer R, Ng S. Differential Recall as a Source of Bias in Epidemiologic Research. *J Clin Epidemiol* 1990;43:1337-41.

36. Knottnerus JA, Tugwell P. Selection-Related Bias, An Ongoing Concern in Doing and Publishing Research. *J Clin Epidemiol* 2014;67:1057-8. http://www.jclinepi.com/article/S0895-4356(14)00332-1/pdf (Accessed: 15 September 2015).

37. van de Mortel TF. Faking it: Social Desirability Response Bias in Self-Report Research. *Aust J Adv Nurs* 2008;25:40-48. http://www.ajan.com.au/ajan_25.4.html (Accessed: 12 February 2017).

38. Kadam P, Bhalerao, S. Sample Size Calculation. *Int J Ayurveda Res* 2010;1:55-57.

For peer review only

For peer review only - http://bmjopen.bmj.com/site/about/guidelines.xhtml

APPENDIX A: ADAPTED STUDY QUESTIONNAIRE

HEPATITIS B VACCINATION QUESTIONNAIRE

For Official Use Only

Researcher…………………………………….. Questionnaire no: ☐

Date of Data Collection (DD/MM/YY)……………………….

IMPLIED CONSENT (Please read before completing questionnaire): Having gone through the research information contained in the participant information sheet, by completing this questionnaire you are consenting to participate in the study. If you do not wish to complete the questionnaire, please put the blank version into the envelope and I will collect it with all other questionnaires. Thank you.

Please only complete the questionnaire if you are aged 18 years and above and have at least 6 months of service with FRSC

Note: This questionnaire is anonymous; please do not write your name on it. Kindly give an answer to all the questions as it pertains to you and please answer as truthfully as you can.

Please check (✔) only the box that most correctly answers the question, making sure you make only one selection for each question except where otherwise indicated.

Section A: Demographic Questions

1. What is your sex?

☐ Male

☐ Female

For peer review only

2. What was your age on your last birthday?

- ☐ 18 to 29 years
- ☐ 30 to 39 years
- ☐ 40 to 49 years
- ☐ 50 years and above

Section B: Employment History

3. How long have you worked with Federal Road Safety Corps (FRSC)?

- ☐ 6 months to 2 years
- ☐ 3 years to 10 years
- ☐ 11 years to 19 years
- ☐ 20 years and above

4. What is your cadre?

- ☐ Officer
- ☐ Marshal Inspectorate
- ☐ Road Marshal Assistant

5. Please check (✔) the box below your rank

ACM	CC	DCC	ACC	CRC	SRC	RC	DRC	ARC

CI	DCI	ACI	PMI	SMI	MI-I	MI-II	MI-III	CRMA	DCRMA	SRMA	RMAI	RMAII	RMAIII

For peer review only

Section C: Perception of Risk of Exposure to Hepatitis B Virus

6. Have you ever heard about hepatitis B virus infection?

- [ ] Yes
- [ ] No

7. How serious do you think being infected with hepatitis B virus is compared to HIV?

- [ ] Less serious than HIV
- [ ] As serious as HIV
- [ ] More serious than HIV
- [ ] I don't know

8. How can someone be infected with hepatitis B virus? (please check (✔) all the correct boxes if your answer is more than one)

- [ ] Through contact with blood of an infected person
- [ ] Through contact with saliva of an infected person
- [ ] Through contact with sweat of an infected person
- [ ] Through contact with body fluid contaminated by blood of an infected person
- [ ] I don't know

For peer review only

9. How much do you think your work with FRSC exposes you to the risk of contracting hepatitis B virus infection?

- ☐ No risk of exposure
- ☐ Low risk of exposure
- ☐ Moderate risk of exposure
- ☐ High risk of exposure
- ☐ I don't know

Section D: Hepatitis B vaccination Knowledge and Status

10. Have you ever heard about hepatitis B vaccination?

- ☐ Yes
- ☐ No

11. How effective do you think hepatitis B vaccination is in protecting someone against hepatitis B virus infection?

- ☐ Not effective
- ☐ Slightly effective
- ☐ Very effective
- ☐ I don't know

12. Have you ever received hepatitis B vaccination?

- ☐ Yes
- ☐ No

If your answer to question 12 is 'No', answer question 13; if it is 'Yes', go to question 14

For peer review only

13. Why have you not received hepatitis B vaccination? (Please check (✔) all the correct boxes if your answer is more than one)

- ☐ I am not aware of hepatitis B vaccination
- ☐ I do not know where to go and receive it
- ☐ I don't have time
- ☐ It is expensive
- ☐ I don't see the need
- ☐ I am afraid of contracting the virus from the vaccine
- ☐ Others (please state)……………………………………………………………………………

14. If your answer to question 12 is 'Yes', how many doses of hepatitis B vaccine have you received?

- ☐ 1 dose
- ☐ 2 doses
- ☐ 3 doses
- ☐ More than 3 doses

15. When did you receive the last dose of hepatitis B vaccine?

- ☐ Less than 1 month ago
- ☐ 1 month to 3 months ago
- ☐ 4 months to 6 months ago
- ☐ More than 6 months ago

For peer review only

16. What do you think is the recommended full dose of hepatitis B vaccine?

- ☐ 1 dose
- ☐ 2 doses
- ☐ 3 or more doses
- ☐ I don't know

17. How long does a full dose of hepatitis B vaccine protect someone?

- ☐ Less than 1 year
- ☐ 1 year to 5 years
- ☐ 6 years to 10 years
- ☐ 11 years to 19 years
- ☐ 20 years or more
- ☐ I don't know

Thank you for your time!

For peer review only

APPENDIX B: Knowledge Scoring Table

Scoring Table: HBV (A) and HBVc (B) Knowledge Questions and Scoring Pattern

**A. Question**	**Options checked**	**Score**
Have you heard about hepatitis B virus infection? (**one option**)	Yes (✔)<br><br>No (✔)	1<br><br>0
How serious do you think being infected with hepatitis B virus is compared to HIV? (**one option**)	Less serious than HIV (✔)<br><br>As serious as HIV (✔)<br><br>More serious than HIV (✔)<br><br>I don't know (✔)	0<br><br>0<br><br>1<br><br>0
How can someone be infected with hepatitis B virus? (please check (✔) all the correct boxes if your answer is more than one)	Through contact with blood of an infected person (✔)<br><br>Through contact with saliva of an infected person (**blank**)*<br><br>Through contact with sweat of an infected person (**blank**)*<br><br>Through contact with body fluid contaminated by blood of an infected person (✔)<br><br>I don't know (✔)	1<br><br>1<br><br>1<br><br>1<br><br>0 for all options
Maximum HBV knowledge Score<br>Minimum HBV knowledge Score		6 of 6<br>0 of 6
**B. Question**	**Options**	**Score**
Have you ever heard about hepatitis B vaccination? (**one option**)	Yes (✔)<br><br>No (✔)	1<br><br>0
How effective do you think hepatitis B vaccination is in protecting someone against hepatitis B virus infection? (**one option**)	Not effective (✔)<br><br>Slightly effective (✔)<br><br>Very effective (✔)<br><br>I don't know (✔)	0<br><br>0<br><br>1<br><br>0
What do you think is the recommended full dose of hepatitis B vaccine? (**one option**)	1 dose (✔)<br><br>2 doses (✔)<br><br>3 or more doses (✔)<br><br>I don't know (✔)	0<br><br>0<br><br>1<br><br>0
How long does a full dose of hepatitis B vaccine protect	Less than 1 year (✔)	0

For peer review only

A. Question	Options checked	Score
someone? (**one option**)	1 year to 5 years (✔)	0
	6 years to 10 years (✔)	0
	11 years to 19 years (✔)	0
	20 years or more (✔)	1
	I don't know (✔)	0
Maximum HBVc knowledge score		4 of 4
Minimum HBVc knowledge score		0 of 4

***** HBV can be found in saliva but the concentration is very low compared to blood; direct injection through bites is required to transmit the virus via this medium.[1,2] Transmission has not been observed through sweat.[1]

References:

1. Canadian Center for Occupational Health and Safety (CCOHS). Hepatitis B. *OSH Facts Sheet* 2014. http://www.ccohs.ca/oshanswers/diseases/hepatitis_b.html (Accessed: 27th July 2015).
2. Centers for Disease Control and Prevention (CDC). When Someone Close to You has Viral Hepatitis. *Division of Viral Hepatitis* 2010. http://www.cdc.gov/hepatitis/HBV/PDFs/HepBWhenSomeoneClose.pdf (Accessed: 26th June 2015).

For peer review only

Appendix C: Frequency of Available Data and Missing Data from Completed Questionnaires, Federal Road Safety Corps, Kaduna Sector Command, Nigeria, June-July, 2015

Variable	Valid Sample Size	Available Data	Percentage	Missing Data	Percentage
Sex	341	327	95.9	14	4.1
Age	341	338	99.1	3	0.9
Duration of Service	341	339	99.4	2	0.6
Cadre	341	341	100	0	0
Ever heard of HBV infection?	341	340	99.7	1	0.3
Seriousness of HBV compared to HIV	341	335	98.2	6	1.8
Route of Transmission of HBV	341	337	98.8	4	1.2
Perception of Risk of exposure to HBV	341	340	99.7	1	0.3
Ever heard of hepatitis B vaccination?	341	336	98.5	5	1.5
Effectiveness of hepatitis B vaccination	341	334	97.9	7	2.1
Ever received hepatitis B vaccination?	341	325	95.3	16	4.7
Number of doses received	198	198	100	0	0
Recommended full dose of hepatitis B vaccine	341	308	90.3	33	9.7
Duration of protection from full-dose HBVc	341	312	91.5	29	8.5

STROBE 2007 (v4) Statement—Checklist of items that should be included in reports of *cross-sectional studies*

Section/Topic	Item #	Recommendation	Reported on page #
**Title and abstract**	1	(*a*) Indicate the study's design with a commonly used term in the title or the abstract	1
		(*b*) Provide in the abstract an informative and balanced summary of what was done and what was found	3,4
**Introduction**			
Background/rationale	2	Explain the scientific background and rationale for the investigation being reported	5-7
Objectives	3	State specific objectives, including any prespecified hypotheses	7
**Methods**			
Study design	4	Present key elements of study design early in the paper	7
Setting	5	Describe the setting, locations, and relevant dates, including periods of recruitment, exposure, follow-up, and data collection	7-10
Participants	6	(*a*) Give the eligibility criteria, and the sources and methods of selection of participants	9,10
Variables	7	Clearly define all outcomes, exposures, predictors, potential confounders, and effect modifiers. Give diagnostic criteria, if applicable	11-12
Data sources/ measurement	8*	For each variable of interest, give sources of data and details of methods of assessment (measurement). Describe comparability of assessment methods if there is more than one group	9-12
Bias	9	Describe any efforts to address potential sources of bias	9-13
Study size	10	Explain how the study size was arrived at	9
Quantitative variables	11	Explain how quantitative variables were handled in the analyses. If applicable, describe which groupings were chosen and why	10-13
Statistical methods	12	(*a*) Describe all statistical methods, including those used to control for confounding	10-13
		(*b*) Describe any methods used to examine subgroups and interactions	NA
		(*c*) Explain how missing data were addressed	13
		(*d*) If applicable, describe analytical methods taking account of sampling strategy	10-13
		(*e*) Describe any sensitivity analyses	NA
**Results**			

For peer review only

Participants	13*	(a) Report numbers of individuals at each stage of study—eg numbers potentially eligible, examined for eligibility, confirmed eligible, included in the study, completing follow-up, and analysed	13-18
		(b) Give reasons for non-participation at each stage	13
		(c) Consider use of a flow diagram	NA
Descriptive data	14*	(a) Give characteristics of study participants (eg demographic, clinical, social) and information on exposures and potential confounders	13,14
		(b) Indicate number of participants with missing data for each variable of interest	13
Outcome data	15*	Report numbers of outcome events or summary measures	16
Main results	16	(*a*) Give unadjusted estimates and, if applicable, confounder-adjusted estimates and their precision (eg, 95% confidence interval). Make clear which confounders were adjusted for and why they were included	16-18
		(*b*) Report category boundaries when continuous variables were categorized	12
		(*c*) If relevant, consider translating estimates of relative risk into absolute risk for a meaningful time period	NA
Other analyses	17	Report other analyses done—eg analyses of subgroups and interactions, and sensitivity analyses	NA
**Discussion**			
Key results	18	Summarise key results with reference to study objectives	19
Limitations	19	Discuss limitations of the study, taking into account sources of potential bias or imprecision. Discuss both direction and magnitude of any potential bias	22-23
Interpretation	20	Give a cautious overall interpretation of results considering objectives, limitations, multiplicity of analyses, results from similar studies, and other relevant evidence	19-22
Generalisability	21	Discuss the generalisability (external validity) of the study results	22,23
**Other information**			
Funding	22	Give the source of funding and the role of the funders for the present study and, if applicable, for the original study on which the present article is based	2

*Give information separately for cases and controls in case-control studies and, if applicable, for exposed and unexposed groups in cohort and cross-sectional studies.

Note: An Explanation and Elaboration article discusses each checklist item and gives methodological background and published examples of transparent reporting. The STROBE checklist is best used in conjunction with this article (freely available on the Web sites of PLoS Medicine at http://www.plosmedicine.org/, Annals of Internal Medicine at http://www.annals.org/, and Epidemiology at http://www.epidem.com/). Information on the STROBE Initiative is available at www.strobe-statement.org.

For peer review only
